# Developmental Stage, Muscle and Genetic Type Modify Muscle Transcriptome in Pigs: Effects on Gene Expression and Regulatory Factors Involved in Growth and Metabolism

**DOI:** 10.1371/journal.pone.0167858

**Published:** 2016-12-09

**Authors:** Miriam Ayuso, Almudena Fernández, Yolanda Núñez, Rita Benítez, Beatriz Isabel, Ana I. Fernández, Ana I. Rey, Antonio González-Bulnes, Juan F. Medrano, Ángela Cánovas, Clemente J. López-Bote, Cristina Óvilo

**Affiliations:** 1 Departamento de Producción Animal, Facultad de Veterinaria, Universidad Complutense de Madrid, Madrid, Spain; 2 Departamento de Mejora Genética Animal, INIA, Madrid, Spain; 3 Comparative Physiology Group. SGIT-INIA, Madrid, Spain; 4 Department of Animal Science, University of California Davis, Davis, California, United States of America; Universitat de Lleida, SPAIN

## Abstract

Iberian pig production includes purebred (IB) and Duroc-crossbred (IBxDU) pigs, which show important differences in growth, fattening and tissue composition. This experiment was conducted to investigate the effects of genetic type and muscle (*Longissimus dorsi* (LD) *vs Biceps femoris* (BF)) on gene expression and transcriptional regulation at two developmental stages. Nine IB and 10 IBxDU piglets were slaughtered at birth, and seven IB and 10 IBxDU at four months of age (growing period). Carcass traits and LD intramuscular fat (IMF) content were measured. Muscle transcriptome was analyzed on LD samples with RNA-Seq technology. Carcasses were smaller in IB than in IBxDU neonates (p < 0.001), while growing IB pigs showed greater IMF content (p < 0.05). Gene expression was affected (p < 0.01 and Fold change > 1.5) by the developmental stage (5,812 genes), muscle type (135 genes), and genetic type (261 genes at birth and 113 at growth). Newborns transcriptome reflected a highly proliferative developmental stage, while older pigs showed upregulation of catabolic and muscle functioning processes. Regarding the genetic type effect, IBxDU newborns showed enrichment of gene pathways involved in muscle growth, in agreement with the higher prenatal growth observed in these pigs. However, IB growing pigs showed enrichment of pathways involved in protein deposition and cellular growth, supporting the compensatory gain experienced by IB pigs during this period. Moreover, newborn and growing IB pigs showed more active glucose and lipid metabolism than IBxDU pigs. Moreover, LD muscle seems to have more active muscular and cell growth, while BF points towards lipid metabolism and fat deposition. Several regulators controlling transcriptome changes in both genotypes were identified across muscles and ages (*SIM1*, *PVALB*, *MEFs*, *TCF7L2* or *FOXO1*), being strong candidate genes to drive expression and thus, phenotypic differences between IB and IBxDU pigs. Many of the identified regulators were known to be involved in muscle and adipose tissues development, but others not previously associated with pig muscle growth were also identified, as *PVALB*, *KLF1* or *IRF2*. The present study discloses potential molecular mechanisms underlying phenotypic differences observed between IB and IBxDU pigs and highlights candidate genes implicated in these molecular mechanisms.

## Introduction

Modern pig production is mostly based on extensively selected breeds, which show optimized productivity and efficiency [1]. However, in the Mediterranean area these commercial breeds coexist with local breeds, used for the production of unique high-quality traditional pork products. These breeds, usually known as fatty-pigs, are smaller in size, have not undergone intense genetic selection and are less productive than modern breeds [2]. Moreover, due to the traditional rearing system, under extensive free-range conditions, they are exposed to harsh environments and seasonal variations in food availability (associated with the development of a thrifty genotype) [3].

The Iberian pig is the most representative Mediterranean traditional breed, and it has an important commercial value based on high quality dry-cured products in terms of consumers’ health and acceptance [4]. Iberian pig shows special growth, fattening and meat characteristics, mainly as a consequence of its particular high fat deposition and desaturation potential, high food intake associated with leptin resistance [5, 6] and the elevated age at which those pigs are slaughtered [7, 8]. Iberian pigs require a long productive cycle (12–18 months) to develop desirable characteristics expected from Iberian pork products. It has been reported that parameters such as intramuscular fat (IMF) content, fatty acids (FA) profile or redness influence meat quality and improve as pigs mature [8–12].

Due to the low productivity of this breed and to the long time required to obtain Iberian products, the Duroc breed has been introduced as a terminal sire to improve reproductive and growth performances and primal cuts yield. However, the introduction of Duroc genetics is associated with a decrease in meat quality, mainly determined by a decrease in IMF and monounsaturated fatty acids (MUFA) contents [13].

Intramuscular fat content and fatty acid composition are main factors affecting meat quality and strongly depend on genetic type, diet, anatomical location and age [10, 14, 15]. Both the number and size of adipocytes within muscle fibers determine intramuscular fat content. During prenatal development and immediately after birth, preadipocyte differentiation is a very active process that slows down with animal growth [16]. Later in growth, hypertrophy is the most important issue affecting IMF content, although hyperplasia is maintained in the adult animal to a lesser extent [17]. Hypertrophy is driven by triglycerides accumulation in mature adipocytes. The ratio of synthesis (lipogenesis) and degradation (lipolysis) determines the amount of triglycerides stored in these cells. Due to the different timing of those two processes (hyperplasia and hypertrophy), sequential studies across growing stages are required for understanding the set of molecular mechanisms driving fat accretion in Iberian pigs Moreover, fat accumulation also depends on the anatomical location. Several studies reported differences in lipid composition (probably associated with muscle fiber composition) [15, 18], oxidative properties and IMF content [19, 20] across muscles.

Due to the importance of lipid synthesis and accumulation in meat quality [21], new interest has arisen towards the understanding of genetic mechanisms underlying such processes. With this goal, some studies based on microarray technology investigated transcriptome differences between genotypes, such as Iberian vs. Large White or Duroc in endocrine tissues [22]. On the other hand, several studies have addressed the effect of the developmental stage (prenatally and postnatally) on muscle transcriptome [23–25] and the effect of muscle type on transcriptome and proteome, showing important functional differences [26, 27]. For example, 15–30% of proteome has been reported to differ between *Longissimus dorsi* (LD) and *Biceps femoris* (BF) muscles [27, 28]. Both LD and BF muscles are of high economic relevance in the Iberian pig industry. So far, LD has been examined in more detail and more frequently but, due to the aforementioned differences, the usefulness of the joint analysis of different muscles is evident, as proposed by Sobol *et al*. [26].

Previous studies focused on transcriptomic differences between pure Iberian and Duroc-crossbred Iberian pigs using microarray technology in the loin [29] and RNA-Seq technology in BF muscle [30]. However, this is the first RNA-Seq technology-based study assessing genetic differences between genotypes, developmental stages and muscle types aimed at improving the knowledge of the genetic and metabolic basis of meat quality and productive traits in Iberian pigs.

A previous study analyzing BF transcriptome in IB pigs showed phenotypic and transcriptomic differences associated with growth and meat quality [30]. The present study arises from that previous findings aiming to: 1) Address the effects of genetic type, muscle, developmental stage and their interactions on phenotypic parameters; 2) Evaluate changes in muscle gene expression conditional on growth stage, genetic type and muscle, that might be responsible for the observed phenotypic differences and identify pathways and networks in which those genes are involved; 3) Identify transcription factors affecting gene expression in order to establish potential new candidate genes affecting productive parameters and meat quality in purebred and Duroc-crossbred Iberian pigs, which could become targets for future studies as genetic markers for selection.

## Materials and Methods

### Ethics statement

Animal manipulations were done in compliance with the regulations of the Spanish Policy for Animal Protection RD1201/05, which meets the European Union Directive 86/609 about the protection of animals used in research. The experiment was specifically assessed and approved (report CEEA 2010/003) by the INIA (Instituto Nacional de Investigación y Tecnología Agraria y Alimentaria) Committee of Ethics in Animal Research, which is the named Institutional Animal Care and Use Committee (IACUC) for INIA.

### Animals and sample collection

Twenty-two pure Iberian sows raised in the same commercial farm were utilized at their third gestation cycle. All females were managed in the same conditions. Eleven sows were mated to Iberian boars and eleven to Duroc boars. Ten litters were employed for the sampling at birth (5 for purebreds and 5 for crossbreds) and twelve for sampling at growing stage (6 for purebreds and 6 for crossbreds). At birth, nine IB and 10 IBxDU male piglets were randomly selected from the ten litters (two from each litter with the exception of one litter, which provided only a single Iberian male for the study). The piglets were sampled immediately after euthanasia performed by stunning and exsanguination in compliance with RD1201/05 standard procedures. The pigs coming from the remaining litters were subject to standard productive management, being both groups fed ad-libitum the same barley-wheat commercial growing diet up to four months of age (growing period), when seven IB and 10 IBxDU were randomly selected (two from each one of 5 litters and seven litters providing only one male each) were slaughtered as previously described. Blood samples were collected from newborns and growing pigs in sterile heparin blood vacuum tubes (Vacutainer Systems Europe, Meylan, France). Immediately after recovery, the blood was centrifuged at 1500 g for 15 min and the plasma was separated and stored into polypropylene vials at −20°C until assayed for determination of glucose and lipids metabolism-indicating parameters. After blood collection, pigs were slaughtered. Several body measures were obtained with a tape measure: total body length (from the rostral edge of the snout to the tail insertion), ham length (from the anterior edge of the *Symphysis pubica* to the *articulatio tarsi)*, total length of anterior and posterior limbs (from the distal edge of the hooves to the proximal edge of the *scapula* or *Symphysis pubica*, respectively); and thoracic, abdominal and ham circumferences. Carcasses were weighted and samples from LD muscle in newborn and growing pigs were vacuum-packed in low-oxygen permeable film and kept frozen at –20°C until fatty acid composition analysis. Prior to fatty acid analysis, muscle samples were freeze dried for two days in a lyophilizer (Lyoquest, Telstar, Tarrasa, Spain) and ground in a Mixer Mill MM400 (Retsch technology, Haan, Germany) until muscle was completely powdered. For transcriptomic analysis, LD samples were immediately frozen in liquid nitrogen and maintained at –80°C until RNA extraction.

Plasma parameters for glucose (glucose and fructosamine) and lipid metabolism (triglycerides, total cholesterol, high-density lipoprotein cholesterol, HDL-c, and low-density lipoprotein cholesterol, LDL-c) plasmatic levels were measured with a clinical chemistry analyzer (Saturno 300 plus, Crony Instruments s. r. l., Rome, Italy).

Plasma metabolites and carcass data from newborn pigs were analyzed in a previous study [30].

### Tissue composition analysis

*Longissimus dorsi* muscle IMF content was quantified using the method proposed by Segura *et al*. [31] based on gravimetrical determination of lipid content. Fatty acid methyl esters (FAMEs) were identified by gas chromatography as described by López-Bote *et al*. [32] using a Hewlett Packard HP-6890 (Avondale, PA, USA) gas chromatograph equipped with a flame ionization detector and a capillary column (HP-Innowax, 30 m × 0.32 mm i.d. and 0.25 μm polyethylene glycol-film thickness). Results were expressed as grams per 100 grams of detected FAMEs.

### Statistical analyses of tissue composition

Phenotypic data were analyzed using the general linear model (GLM) procedure using SAS version 9.2 (SAS Inst. Inc., Cary, NC; 2009). The mean and genetic type/muscle/developmental stage were considered as systematic effects, and residual effects as random. Carcass weight was used as covariate when it was significant and removed from the model when it was not. The animal was the experimental unit for all analysis. The results were considered to be significant at *p-*value < 0.05.

### Transcriptomic analysis

#### RNA extraction

A total of 24 animals were randomly chosen among the available ones, assuring the representation of all available litters, and used to perform transcriptomic analysis (6 animals of each genetic type at each studied age). Total RNA was extracted from 50-100mg samples of LD muscle using the RiboPure TM of High Quality total RNA kit (Ambion, Austin, TX, USA) following the manufacturer’s recommendations. RNA was quantified using a NanoDrop-100 spectrophotometer (NanoDrop Technologies, Wilmington, DE, USA). The quality of the RNA was evaluated using the RNA Integrity Number (RIN) value from the Agilent 2100 Bioanalyzer device (Agilent technologies, Santa Clara, CA, USA). The RIN values ranged from 7.8 to 9.8.

#### Library construction and RNA sequencing

Sequencing libraries were made using the mRNA-Seq sample preparation kit (Illumina Inc., Cat. # RS-100-0801) according to manufacturer’s protocol. Each library was sequenced using TruSeq SBS Kit v3-HS, in paired end mode with the read length 2x76 bp on a on a HiSeq2000 sequence analyzer (Illumina, Inc). Images from the instrument were processed using the manufacturer’s software to generate FASTQ sequence files.

#### Mapping and assembly

Sequence reads were analyzed using CLC Bio Genomic workbench software 7.0 (CLC Bio, Aarhus, Denmark). Quality control analysis was performed using the NGS quality control tool, which assesses sequence quality indicators based on the FastQC-project (FastQC-project ). Quality was measured taking into account sequence-read lengths and base-coverage, nucleotide contributions and base ambiguities, quality scores as emitted by the base caller and over-represented sequences [33]. All the samples analyzed passed all the QC parameters having the same length (76 bp), 100% coverage in all bases, 25% of A, T, G and C nucleotide contributions, 50% GC on base content and less than 0.1% over-represented sequences. A hierarchical clustering of the samples was also performed to detect samples that might have been wrongly allocated to a group, samples of unintended or unclean tissue composition or samples for which the processing has gone wrong. Sequence paired-end reads (76bp) were assembled against the annotated Sscrofa10.2 reference genome (http://www.ncbi.nlm.nih.gov/genome/?term=sus+scrofa) using the genome, annotated genes and mRNA tracks. Data was normalized by calculating the ‘fragments per kilo base per million mapped reads’ (FPKM) for each gene [34].

#### Differential expression analysis

The statistical analysis was performed using the total exon reads as expression values by the Empirical analysis of differential gene expression tool (CLC Bio, Aarhus, Denmark). This tool is based on the EdgeR Bioconductor package [35] and uses count data (i.e. total exon reads) and the same normalization method (trimmed mean of M-values, TMM [35]) for the statistical analysis. Genes were filtered according to two criteria: a minimum mean group expression greater than 0.5 FPKM in at least one group and a Fold-Change (FC) of the expression differences between genotypes, muscles and developmental stages equal or higher to 1.5. Finally, those genes with a *p-*value ≤ 0.01, corresponding to a false discovery rate (FDR) value ≤ 0.14 in newborns and ≤ 0.20 in growing pigs, were considered as differentially expressed (DE). Gene expression data of BF muscle was previously obtained and analyzed [30] following the same criteria. Three different effects were independently assessed in the differential expression study: developmental stage effect on LD muscle transcriptome, genetic type effect on LD muscle transcriptome (at both ages independently); and muscle type effect (LD vs BF) at birth.

#### Results validation by RT qPCR

RNA obtained from the 24 animals used in the RNA-Seq assay was utilized to perform the technical validation of the differential expression of some genes that were either affected by the developmental stage, the muscle or the genetic type. Moreover, RNA obtained from all the available animals (16 IB and 20 IBxDU) was used to quantify expression differences by qPCR [30]. The expression of 5 genes: *MTUS2*, *ALDH2*, *ADAMTS8*, *HDAC9* and *SIM1 (Single-Minded Family BHLH Transcription Factor 1)*) was quantified employing methods previously described [30]. The *GAPDH*, *TBP*, *B2M* and *ACTB* genes were selected as the most stable endogenous genes between the different studied conditions to normalize the data.

The technical validation was performed by studying the Pearson correlation between the expression values obtained from RNAseq data (FPKM) and the normalized gene expression data obtained by RT qPCR, as previously described [30]. To validate the global RNA-Seq results, the concordance correlation coefficient (CCC) [36] was calculated between the FC values estimated from RNA-Seq and qPCR expression measures for the 5 genes analyzed by the two technologies (RNA-Seq and qPCR).

#### Systems biology study

The biological interpretation of the DE genes was performed using two complementary approaches in order to identify: 1) enriched pathways and networks involving the DE genes, and 2) potential regulators causing the observed changes in gene expression.

Ingenuity Pathway Analysis, (IPA) (Ingenuity Systems, Qiagen, California) software was utilized to identify and characterize biological functions, gene networks and canonical pathways affected by the DE genes. The IPA Canonical Pathways Analysis identified the pathways from the Ingenuity Pathways Analysis library of canonical pathways that were most significant in our dataset. The significance of the association between the dataset and the canonical pathway was measured with Fischer’s exact test, to calculate a P‒value determining the probability that the association between the genes in the dataset and the canonical pathway is explained by chance alone.

Regulatory transcription factors (TRF), which could potentially affect the DE genes in the dataset were also studied by following complementary approaches. First, RIF1 and RIF2 metrics [37, 38] were calculated for the whole set of DE genes obtained conditional on developmental stage (5,812 genes), muscle type (135 genes) and genetic type at birth (261 genes) and at the growing period (113 genes). Candidate TRFs in pigs were obtained from Animal TFDB ("http://www.bioguo.org/AnimalTFDB/BrowseAllTF.php?spe=Susscrofa"). A total of 1,038 TRF were retrieved. Among them, 734, 739, 655 and 738 showed expression values greater than 0.5 FPKM in at least one experimental group when analyzing the developmental stage, genetic type (at birth and at growth) and the muscle type effects, respectively. Those TRFs were thus used in the RIFs metrics approach.

The RIF1 and RIF2 values were computed for the *i*^th^ TRF as follows:

$${RIF1}_{i}=\frac{1}{n_{de}}\sum_{j=1}^{j=n_{de}}{â}_{j}{xd}_{j}{\left({r1}_{ij}-{r2}_{ij}\right)}^{2}$$

and

$${RIF2}_{i}\;=\;\frac{1}{n_{de}}\sum_{j\;=\;1}^{j\;=\;n_{de}}\left[{\left({e1}_{j}{xr1}_{ij}\right)}^{2}-{\left({e2}_{j}{xr2}_{ij}\right)}^{2}\right]$$

Where n_de_ is the number of DE genes, aj and dj the estimated average expression and differential expression of the *j*^*th*^ DE gene, r1ij and r2ij the co-expression correlation between the *i*^*th*^ TRF and the *j*^*th*^ DE gene in each one of the genetic types and being e1j and e2j the expression of the *j*^*th*^ gene in each genetic type [39]. Both RIF measures for each analyzed TRF were transformed to standardized *z*-scores by subtracting the mean and dividing by its standard deviation. We identified relevant TRF as those with extreme RIF z-scores according to the corresponding confidence intervals (CI) calculated by bootstrap. In each iteration of bootstrapping, a set of *n*_*de*_ *= number of DE genes* were randomly selected from the total expressed genes, and the RIF1 and RIF2 z-scores of the expressed TRF list were calculated for each studied effect. The procedure was repeated 10,000 times for each scenario to obtain the corresponding 95 and 99% CI intervals of both z-scores.

Complementarily, IPA software was used to identify and characterize potential regulators using two different tools, the *upstream regulators* and the *regulators* tools. Both of them identify known regulators that may affect the expression of the data set of DE genes. IPA-identified regulators include genes, but also other molecules such as drugs. Thus, out of the identified regulators, only genes that were also included in the RIFs metrics candidate TRF list were considered (genes included in the animal TFDB and with expression values higher than 0.5 FPKM in at least one experimental group).

Using the information obtained from the TRF study, an additional search for enriched pathways and networks was carried out with IPA software considering both, DE genes and TRF conditional on developmental stage, muscle and genetic type.

## Results

The transcriptome of LD muscle was studied at two different developmental stages, birth (mean live weight of 1.5 (SD = 0.4) kg) and growth (mean live weight of 64.9 (SD = 10.1) kg). Moreover, the BF muscle transcriptome was previously assessed at birth in the same newborn pigs [30]. The experimental design allowed the study of several main effects regarding muscle transcriptome: developmental stage, genetic type and tissue effects. The biggest transcriptome change was observed between the two studied ages, involving more than 5,800 DE genes (Table 1), followed by the genetic type effect, responsible for differential expression of 261 genes at birth and 113 at the growing stage and the tissue effect, with 135 DE genes between LD and BF muscles (Table 1).

**Table 1:** Differentially expressed (DE) genes as affected by the three studied main effects.

	Total DE genes	Upregulated genes	
		Birth	four months
Developmental stage effect (LD)	5812	3290	2522
		IBxDU^1^	IB^2^
Genotype effect at birth (LD)	261	131	130
Genotype effect at four months (LD)	113	25	88
		BF^3^	LD^4^
Muscle effect (birth)	135	52	83

^1^ IBxDU = Iberian x Duroc crossbred pigs

^2^ IB = Purebred Iberian pigs

^3^ BF = *Biceps femoris* muscle

^4^ LD = *Longissimus dorsi* muscle

### Phenotypic results

Developmental stage and genetic type significantly affected carcass characteristics, plasma biochemical parameters and meat quality traits, such as IMF and fatty acid profile (Table 2). Pure Iberian and crossbred piglets had an average of 1.2 and 1.8 kg birth-weight, respectively (p<0.001; Table 2). Genetic type affected all the carcass phenotypic parameters at this early age as reported in a previous study employing the same newborn animals [30]: IBxDU neonates were bigger and heavier (p < 0.001) than IB newborns. On the other hand, growing IB and IBxDU pigs slaughtered at 59.4 and 68.6 kg live weight, respectively, showed no significant difference in body weight or size, although ham weight and perimeter were higher in IBxDU than in IB pigs (p = 0.039 and p = 0.034, respectively). A significant interaction between developmental stage and genotype was observed for ham weight and circumference measures.

**Table 2:** Effect of genotype, developmental stage and their interaction on phenotype of pure and crossbred Iberian pigs.

Genetic type (GT)									Stage	GT*Stage
Birth					Growing					
Carcass traits	IBxDU^1^	IB^2^	SEM^3^	p-value	IBxDU^4^	IB^5^	SEM	p-value	p-value	p-value
Live weight	1.77	1.21	0.07	< .001	68.60	59.40	2.41	0.084	< .001	0.054
Carcass weight	1.41	0.96	0.05	< .001	56.22	48.60	2.17	0.109	< .001	0.074
Carcass length	40.20	35.50	0.54	< .001	121.11	116.17	1.54	0.140	< .001	0.936
Thorax circumference	25.15	22.06	0.39	0.001	89.17	86.00	1.11	0.186	< .001	0.974
Abdomen circumference	18.90	17.28	0.38	0.049	77.28	78.17	1.21	0.724	< .001	0.288
Ham weight	0.16	0.11	0.01	< .001	7.56	6.23	0.29	0.040	< .001	0.017
Ham length	7.45	6.33	0.14	< .001	25.89	24.33	0.36	0.052	< .001	0.541
Ham circumference	12.55	10.89	0.23	0.002	67.83	59.42	1.74	0.034	< .001	0.040
Lipid and glucose metabolism-related plasma indicators										
Cholesterol	62.2	102.4	5.60	0.003	107.6	95.1	3.86	0.136	0.144	0.031
LDL^6^	42.2	45.8	4.40	0.449	67.4	61.5	2.58	0.280	0.002	0.431
HDL^7^	22.4	41.2	4.25	0.018	30.2	24.0	2.93	0.313	0.452	0.049
TG^8^	30.0	76.7	5.11	< .001	46.4	53.7	4.03	0.439	0.354	0.026
Fructosamine	169.7	133.7	10.37	0.101	221.3	228.0	8.05	0.691	< .0001	0.135
Glucose	132.4	123.4	10.80	0.684	107.8	92.5	2.91	0.023	0.036	0.804
*Longissimus dorsi* muscle main fatty acids composition (g/100 g total fatty acids)										
IMF^9^	2.16	2.32	0.50	0.317	2.87	4.05	0.20	0.026	< .0001	0.655
C14:0	2.77	2.56	0.12	0.406	1.14	1.32	0.08	0.295	< .0001	0.230
C15:1	1.51	1.26	0.09	0.171	0.60	0.33	0.04	0.026	< .0001	0.743
C16:0	26.29	26.12	0.18	0.655	24.56	25.44	0.39	0.114	0.001	0.072
C16:1 n-9	2.13	2.03	0.05	0.547	0.20	0.27	0.02	0.099	< .0001	0.232
C16:1 n-7	5.93	5.40	0.22	0.234	3.20	3.34	0.13	0.938	< .0001	0.357
C17:0	1.78	1.50	0.07	0.064	0.47	0.32	0.03	0.039	< .0001	0.197
C17:1	0.94	0.88	0.05	0.564	0.46	0.31	0.03	0.042	< .0001	0.862
C18:0	10.62	9.43	0.32	0.083	12.91	12.69	0.23	0.787	< .0001	0.130
C18:1 n-9	24.06	26.61	0.80	0.129	41.39	42.42	0.49	0.373	< .0001	0.085
C18:1 n-7	6.34	5.82	0.21	0.235	2.67	2.21	0.13	0.051	< .0001	0.623
C18:2 n-6	7.00	8.76	0.56	0.132	7.32	5.36	0.32	0.020	0.040	0.022
C18:3 n-3	0.28	0.25	0.02	0.351	0.18	0.18	0.01	0.839	0.001	0.370
C18:4 n-3	0.13	0.17	0.02	0.351	0.08	0.08	0.00	0.347	0.019	0.222
C20:0	0.36	0.27	0.04	0.320	0.20	0.19	0.01	0.963	0.031	0.532
C20:1 n-9	0.63	0.60	0.05	0.786	0.77	0.78	0.02	0.635	0.007	0.957
C20:2 n-6	0.32	0.33	0.06	0.913	0.61	0.45	0.04	0.040	0.024	0.187
C20:3 n-6	0.63	0.52	0.02	0.017	0.25	0.23	0.02	0.525	< .0001	0.064
C20:4 n-6	5.65	4.97	0.27	0.221	2.02	1.18	0.11	0.007	< .0001	0.578
C22:4 n-6	1.07	0.91	0.05	0.175	0.43	0.54	0.04	0.192	< .0001	0.048
C22:5 n-3	0.36	0.33	0.01	0.254	0.34	0.85	0.04	< .0001	< .0001	< .0001
C22:6 n-3	0.31	0.26	0.04	0.544	0.40	0.99	0.11	0.020	0.003	0.012
∑SFA^10^	42.57	40.46	0.54	0.068	39.32	40.13	0.55	0.179	0.009	0.012
∑MUFA^11^	41.41	42.82	0.50	0.174	49.50	49.01	0.64	0.419	< .0001	0.108
∑PUFA^12^	16.02	16.72	0.44	0.441	11.73	9.71	0.50	0.102	< .0001	0.284
UI^13^	91.19	91.79	0.44	0.441	79.37	81.39	1.58	0.752	< .0001	0.968
∑n-3^14^	1.35	1.22	0.91	0.746	1.00	2.10	0.16	0.004	0.198	0.001
∑n-6^15^	14.67	15.50	0.07	0.324	10.73	7.65	0.47	0.013	< .0001	0.051
∑n-6/∑n-3	11.61	12.88	0.42	0.344	14.31	3.89	1.22	0.003	0.043	< .0001

^1^ IBxDU = Iberian x Duroc crossbred pigs (n = 10)

^2^ IB = Purebred Iberian pigs (n = 9)

^3^ SEM = Standard error of the mean

^4^ IBxDU = Iberian x Duroc crossbred pigs (n = 10)

^5^ IB = Purebred Iberian pigs (n = 7)

^6^ LDL = Low density lipoproteins

^7^ HDL = High density lipoproteins

^8^ TG = Triglycerides

^9^ IMF = Intramuscular fat

^10^ ΣSFA = Sum of saturated fatty acids

^11^ ΣMUFA = Sum of monounsaturated fatty acids

^12^ ΣPUFA = Sum of polyunsaturated fatty acids

^13^ UI = Unsaturation index = 1 × (% monoenoics) +2 × (% dienoics) +3 × (% trienoics) +4 × (% tetraenoics) +5 × (% pentaenoics) +6 × (% hexaenoics)

^14^ Σn3 = Sum of n-3 fatty acids

^15^ Σn6 = Sum of n-6 fatty acids

Purebred IB newborns had significantly greater plasma levels of total and HDL cholesterol, and triglycerides (TG) than IBxDU neonates (Table 2). However, growing IB and IBxDU pigs showed similar cholesterol and TG levels, resulting in a developmental stage*genetic type interaction effect for these parameters. On the other hand, growing pigs differed in plasma glucose: IBxDU showed higher levels than IB pigs at this stage (p = 0.023).

Regarding loin IMF, total content increased over time (p < 0.0001). Genetic type did not affect IMF content in LD muscle (Table 2) of newborn piglets, while IB showed a 41% increase in IMF content with respect to IBxDU pigs at the growing stage (p = 0.026). Developmental stage also affected IMF composition: MUFA content increased and PUFA content decreased over time (p < 0.0001) in both genetic types. However, the SFA content slightly decreased along growth in IBxDU but not in IB pigs, with a significant interaction between genotype and developmental stage (p = 0.012). Also, C20:3 n-6 concentration (p = 0.017) was higher in newborn IBxDU pigs and SFA content showed a trend (p = 0.068) in the same direction. In the growing period, C15:1, C17:0, C17:1, C18:2-n6, C20:2 n-6 and C20:4 n-6 concentrations were significantly higher in IBxDU than in IB pigs (p = 0.026, 0.039, 0.042, 0.020, 0.040 and 0.007, respectively) while C22:5 n-3 (DPA) and C22:6 n-3 (DHA) proportions were higher in IB pigs (p < 0.0001 and p = 0.020, respectively). As a consequence, total n-3 fatty acids content was higher in IB (p = 0.004) and total n-6 and the ratio n-6/n-3 was higher in the loin of growing IBxDU pigs (p = 0.013 and p = 0.003, respectively).

Data on BF phenotype and transcriptome was available for the same newborn animals from a previous study [30]. Thus, the effect of the studied muscle (BF *vs* LD) was assessed in IB and IBxDU neonates (Table 3). *Biceps femoris* and LD muscles showed similar IMF content at this early age (p = 0.158). Differences in total PUFA and n-3 PUFA content were observed, with BF showing higher levels than LD muscle. These differences resulted in a lower n-6/n-3 ratio in BF when compared to LD muscle. When analyzing the global effect of genotype on IMF content and composition of both muscles at birth, higher SFA and lower n-6 PUFA content was observed in IBxDU pigs. No significant interaction was found between the genotype and muscle effects.

**Table 3:** *Longissimus dorsi* (LD) and *Biceps femoris* (BF) muscle characteristics of pure and Duroc-crossbred Iberian piglets at birth.

		Mean			p-value		
	Muscle	IBxDU^1^	IB^2^	SEM^3^	Genetic type (GT)	Muscle	GT*Muscle
∑SFA^4^	LD	42.57	40.79	0.33	0.002	0.182	0.538
	BF	41.66	39.91				
∑MUFA^5^	LD	41.41	42.73	0.32	0.114	0.478	0.653
	BF	41.01	41.77				
∑PUFA^6^	LD	16.02	16.48	0.31	0.057	0.039	0.845
	BF	17.34	18.32				
∑n-3^7^	LD	1.35	1.24	0.04	0.145	< .0001	0.942
	BF	1.77	1.69				
∑n-6^8^	LD	14.67	15.24	0.30	0.034	0.123	0.833
	BF	15.56	16.63				
∑n-6/∑n-3	LD	11.61	12.54	0.36	0.056	0.002	0.892
	BF	8.78	9.96				
IMF^9^	LD	2.16	2.26	0.08	0.060	0.158	0.644
	BF	1.80	2.16				

^1^ IBxDU = Iberian x Duroc crossbred pigs (n = 10)

^2^ IB = Purebred Iberian pigs (n = 9)

^3^ SEM = Standard error of the mean

^4^ ΣSFA = Sum of saturated fatty acids

^5^ ΣMUFA = Sum of monounsaturated fatty acids

^6^ ΣPUFA = Sum of polyunsaturated fatty acids

^7^ Σn3 = Sum of n-3 fatty acids

^8^ Σn6 = Sum of n-6 fatty acids

^9^ IMF = Intramuscular fat

### Transcriptome analysis

#### Mapping results

An average of approximately 84 million sequence reads was obtained for each individual sample and were assembled and mapped to the annotated Sscrofa10.2 genome assembly (22,861 genes). In all samples, 67–77% of the reads were categorized as mapped reads to the porcine reference sequence. The FPKM values were used to establish the total number of genes expressed in the muscle transcriptome (> 0.5 FPKM). Approximately 50% of total porcine annotated genes in the Sscrofa10.2 genome assembly were expressed in the studied samples (an average of 11,506 genes out of 22,861 annotated genes).

#### Effect of developmental stage on *Longissimus dorsi* transcriptome

A total of 3,290 genes were upregulated in LD muscle at birth when compared to four months-old pigs (p < 0.01, FDR < 0.015), with FC ranging from 1.5 to 219 (*IBSP* gene showed the largest expression difference). In four months-old pigs, 2,522 genes were upregulated when compared to newborn pigs. Fold changes (FC) ranged from 1.5 to 273, with several immunoglobulin genes showing the biggest expression changes (S1 Table).

Gene expression differences were functionally interpreted using IPA software to detect enriched pathways (S2 Table) and biological functions (Table 4). Moreover, the regulators study gathered 626 genes potentially affecting expression of the DE genes in the dataset (i.e. *MSTN*, *FOXO3*, *FOXO1*, *MEF2C* or *MEF2D*). Among them, 270 were identified by IPA software, 142 were identified following the RIFs approach and 344 were found DE between developmental stages (S3 Table). Within the selected TRF, eight (*CBX4*, *HSF1*, *JARID2*, *MYC*, *TCF4*, *TEAD4*, *TFEB* and *YBX3*) were identified following the three approaches, being considered as especially relevant regulators.

**Table 4:** Enriched biological functions in the set of DE genes between LD muscle from newborn and growing Iberian pigs

Upregulated at birth			Upregulated at growing stage		
Enriched function	Corrected p-value	z-score^1^	Enriched function	Corrected p-value	z-score
Invasion of cells	2.66E-10	3.93	Organismal death	4.90E-23	6.95
Size of body	2.23E-06	3.58	Growth Failure	1.08E-04	4.94
Transport of molecule	1.78E-08	3.33	Bleeding	1.31E-03	4.93
Invasion of tumor cell lines	1.67E-06	3.18	Perinatal death	6.00E-06	3.92
Cell movement	1.63E-17	3.09	contractility of skeletal muscle	5.25E-04	3.86
Cell movement of tumor cell lines	3.06E-07	3.05	Hypoplasia of organ	2.73E-03	3.26
Apoptosis of fibroblast cell lines	5.99E-08	3.03	Hypoplasia	1.62E-04	3.15
Adhesion of connective tissue cells	1.40E-03	2.94	Dysgenesis	1.21E-04	3.04
Cancer	9.37E-54	2.94	Congenital anomaly of musculoskeletal system	7.19E-05	2.92
Migration of cells	2.73E-03	2.90	Multiple congenital anomalies	1.13E-07	2.91
Migration of tumor cell lines	9.70E-15	2.77	Polymerization of protein	2.01E-05	2.73
Limb development	1.46E-09	2.77	Fibrosis	1.06E-04	2.39
Invasion of tumor	5.45E-07	2.70	Hypoplasia of thorax	1.41E-03	2.25
Proliferation of fibroblasts	2.37E-04	2.59	Autophagy	1.84E-03	2.18
Adhesion of tumor cell lines	4.38E-09	2.58	Anemia	1.49E-03	1.84
Disassembly of filaments	5.25E-07	2.56	Mass of muscle	1.99E-14	1.77
Synthesis of DNA	4.31E-05	2.56	Adhesion of extracellular matrix	1.75E-05	1.73
Invasion of breast cancer cell lines	9.33E-05	2.56	Autosomal recessive disease	1.61E-03	1.72
Synthesis of carbohydrate	2.27E-03	2.52	Mass of skeletal muscle	2.14E-07	1.69
Tumorogenesis of tissue	2.43E-04	2.46	Blood vessel defect	1.28E-03	1.56
Neoplasia of cells	1.28E-03	2.45	Systemic autoinmune syndrome	7.10E-05	1.53
Invasion of tumor cells	1.95E-03	2.40	Dwarfism	2.68E-08	1.46
Organization of cytoplasm	1.68E-47	2.36	Abnormal bone density	2.78E-03	1.45
Metabolism of carbohydrate	3.24E-04	2.36	Dysplasia of skeleton	6.50E-13	1.38
Endocytosis	1.21E-05	2.33	Aneurysm	3.42E-18	1.38
Proliferation of neuronal cells	2.23E-03	2.30	Vascular tumor	1.09E-20	1.33
Organization of cytoskeleton	3.35E-16	2.28	Hemangioma	4.13E-04	1.33
Development of central nervoussystem	4.95E-06	2.26	Hypertension	2.16E-03	1.33
Development of body trunk	3.87E-05	2.22	Hypoglycemia	3.79E-05	1.24
Outgrowth of cells	5.94E-07	2.19	Adhesion of cell-associated matrix	1.86E-03	1.19
Neoplasia of epitelial tissue	2.20E-15	2.18	Ulcer	1.11E-04	1.13
Formation of celular protrusions	1.88E-04	2.18	Chronic inflammatory disorder	1.15E-03	1.06
Microtubule dynamics	1.50E-08	2.16	Replication of virus	9.27E-05	1.01
Behavior	6.31E-04	2.12	Quantity of muscle cells	7.41E-04	0.99
Cell death of fibroblasts	1.05E-47	2.11	Mass of hind limb muscle	7.62E-09	0.91
Proliferation of cells	2.64E-08	2.10	Function of muscle	1.35E-06	0.89
Invasion of malignant tumor	1.02E-13	2.10	Breast or ovarían cancer	3.28E-13	0.86
Development of cardiovascular system	3.94E-04	2.06	Quantity of muscle	5.41E-05	0.86
Cell surface receptor linked signal transduction	3.70E-04	2.01	Abdominal aortic aneurysm	5.06E-04	0.83
Angiogenesis	6.11E-37	2.00	Stabilization of microtubules	1.79E-03	0.82

^1^ Z-score: Predicted activation status of biological function. The higher the value, the more activated the functions is predicted to be.

#### Effect of genetic type on *Longissimus dorsi* transcriptome

The effect of genetic type on LD muscle transcriptome was analyzed at birth and at growing independently, due to the high influence of developmental stage on gene expression. Genetic type effect on gene expression seemed to be stronger at birth, since 261 genes were DE (p < 0.01, FDR < 0.141) at that young age while less than a half, 113 DE genes were identified between IB and IBxDU growing pigs (p < 0.01, FDR < 0.199). Out of the 261 DE genes observed at birth, 130 were upregulated in IB (FC from 1.8 to 25.6) and 131 in IBxDU pigs (FC from 1.8 to 58.5). At growth, 88 genes were upregulated in IB (FC from 2.1 to 390) and 25 in IBxDU pigs (FC from 2.2 to 88.4) (S4 Table).

Biological interpretation of the DE genes with IPA software retrieved enriched pathways and biological functions in IB and IBxDU pigs at both studied developmental stages (Tables 5 and 6, respectively). Moreover, the regulators analysis identified 122 TRF at birth and 62 TRF at growth, potentially regulating the gene expression changes observed between genetic types. As described in the regulators study performed for the developmental stage effect, we considered TRF those that were either DE, identified by IPA software as regulators or identified in the RIFs study (S5 Table). Sixteen of those TRF (such as *ATF4*, *CEBPA*, *MYOD1 (Myogenic Differentiation 1)*, *NFE2L2* or *REL*) were found at both ages. Fifteen regulators (*CREB3L1*, *CREBBP*, *CSRP3*, *FOS (FBJ Murine Osteosarcoma Viral Oncogene Homolog)*, *FOXO1*, *HIRA*, *HSF1*, *KLF1 (Kruppel-Like Factor 1)*, *MEF2C*, *MEF2D*, *MYOG*, *NFE2*, *SF1*, *SOX4* and *TEAD3*) were identified following different approaches at birth and 3 (*EN1*, *IRF2* and *TCF7L2*) at growth. Moreover, a combined analysis performed using IPA software by merging DE genes and TRF datasets revealed the *glucocorticoid receptor signaling* and *adipogenesis* as the most enriched pathways at birth (p = 6.14e-11 and p = 1.41e-9, respectively), while the *aryl hydrocarbon receptor* pathway was the most enriched in growing pigs (p = 2.13e-8). The *PPAR signaling* and the *unfolded protein response* pathways were enriched at both stages (S6 Table).

**Table 5:** Enriched pathways in the set of DE genes conditional on genetic type at birth and at growing.

Birth			Growing		
IB^1^	p-value	Corrected p-value	IB	p-value	Corrected p-value
Role of IL-17A in Psoriasis	2.83E-05	6.60E+00	Serine Biosynthesis	5.57E-09	8.08E-08
PI3K Signaling in B Lymphocytes	6.56E-05	6.60E+00	Superpathway of Serine and Glycine Biosynthesis I	1.29E-08	1.42E-07
ERK5 Signaling	7.65E-05	6.60E+00	Glutathione-mediated Detoxification	1.05E-04	5.14E+00
Melatonin Degradation III	1.87E-04	6.60E+00	LPS/IL-1 Mediated Inhibition of RXR Function	1.09E-04	5.14E+00
Glutamine Biosynthesis I	1.87E-04	6.60E+00	Alanine Degradation III	3.01E-04	5.14E+00
NRF2-mediated Oxidative Stress Response	6.00E-04	4.03E+01	Alanine Biosynthesis II	3.01E-04	5.14E+00
IL-8 Signaling	6.92E-04	4.03E+01	Cell Cycle: G1/S Checkpoint Regulation	2.02E-03	5.14E+00
April Mediated Signaling	1.47E-03	4.03E+01	Cyclins and Cell Cycle Regulation	4.96E-03	5.14E+00
Trehalose Degradation II (Trehalase)	1.81E-03	4.09E+01	Glucose and Glucose-1-phosphate Degradation	6.29E-03	5.14E+00
B Cell Activating Factor Signaling	1.81E-03	4.09E+01	p53 Signaling	1.62E-02	5.14E+00
LXR/RXR Activation	2.80E-03	4.09E+01	Leukotriene Biosynthesis	3.71E-02	5.14E+00
Atherosclerosis Signaling	3.13E-03	6.28E+01	LXR/RXR Activation	4.97E-02	5.14E+00
TNFR1 Signaling	4.68E-03	6.28E+01			
Wnt/Ca+ pathway	9.09E-03	6.57E+01			
Glucocorticoid Receptor Signaling	1.17E-02	6.57E+01			
GDP-glucose Biosynthesis	1.52E-02	2.44E+02			
Glucose and Glucose-1-phosphate Degradation	2.27E-02	3.24E+02			
Growth Hormone Signaling	2.61E-02	4.33E+02			
Melatonin Signaling	2.80E-02	4.41E+02			
UDP-N-acetyl-D-galactosamine Biosynthesis II	3.22E-02	4.41E+02			
Role of Macrophages, Fibroblasts and Endothelial Cells in Rheumatoid Arthritis	3.99E-02	4.41E+02			
Production of Nitric Oxide and Reactive Oxygen Species in Macrophages	4.97E-02	4.41E+02			
IBxDU^2^	p-value	Corrected p-value	IBxDU	p-value	Corrected p-value
AldosteroneSignaling in EpithelialCells	1.11E-10	1.02E-09	Acute Phase Response Signaling	3.90E-05	1.73E-02
ProteinUbiquitinationPathway	1.71E-09	2.09E-08	Retinoate Biosynthesis II	9.68E-05	1.73E-02
Unfoldedprotein response	5.94E-08	1.64E-06	Bupropion Degradation	1.03E-03	4.62E-02
eNOSSignaling	6.94E-06	1.47E-03	Acetone Degradation I (to Methylglyoxal)	1.47E-03	4.62E-02
EndoplasmicReticulum Stress Pathway	1.79E-04	1.91E-01	Retinoate Biosynthesis I	2.02E-03	4.62E-02
PyrimidineRibonucleotidesInterconversion	4.74E-04	1.91E-01	Retinol Biosynthesis	2.80E-03	4.62E-02
Glucocorticoid Receptor Signaling	5.45E-04	1.91E-01	Estrogen Biosynthesis	3.51E-03	4.62E-02
PyrimidineRibonucleotides De Novo Biosynthesis	6.29E-04	1.91E-01	Nicotine Degradation III	5.26E-03	4.62E-02
NeuregulinSignaling	6.29E-04	1.91E-01	Melatonin Degradation I	8.03E-03	4.62E-02
Role of p14/p19ARF in Tumor Suppression	7.27E-04	1.91E-01	Nicotine Degradation II	9.68E-03	4.62E-02
SpermineBiosynthesis	8.41E-04	1.91E-01	IL-17 Signaling	1.10E-02	4.62E-02
AlanineDegradation III	8.41E-04	1.91E-01	GABA Receptor Signaling	1.10E-02	4.62E-02
AlanineBiosynthesis II	8.41E-04	1.91E-01	Superpathway of Melatonin Degradation	1.33E-02	4.62E-02
Hepatic Fibrosis / HepaticStellateCellActivation	2.02E-03	5.58E-01	IL-6 Signaling	4.29E-02	4.62E-02
Spermidine Biosynthesis I	2.02E-03	5.58E-01			
PI3K/AKT Signaling	4.96E-03	1.94E+00			
Serine Biosynthesis	7.10E-03	2.93E+00			
Thioredoxin Pathway	1.17E-02	4.26E+00			
Aryl Hydrocarbon Receptor Signaling	1.17E-02	4.26E+00			
Epithelial Adherens Junction Signaling	1.62E-02	5.32E+00			
Superpathway of Serine and Glycine Biosynthesis I	1.85E-02	5.32E+00			
PCP pathway	2.27E-02	6.37E+00			
Hypoxia Signaling in the Cardiovascular System	2.80E-02	6.37E+00			
Mitotic Roles of Polo-Like Kinase	3.00E-02	6.37E+00			
Tight Junction Signaling	4.62E-02	1.03E+01			

^1^ IB = Purebred Iberian pigs

^2^ IBxDU = Iberian x Duroc crossbred pigs

**Table 6:** Enriched biological functions in the set of DE genes conditional on genetic type, at birth and at growth.

Birth				Growth			
IB^1^							
Function	P-value	Corrected p-value	z-score^2^	Function	p-Value	Corrected p-value	z-score
Cell death of tumor cell lines	2.49E-06	5.45E-04	-2.82	Size of body	7.37E-03	4.89E-02	-2.356
Apoptosis	2.05E-09	4.03E-06	-2.60	Quantity of connective tissue	3.22E-03	3.47E-02	-1.987
Apoptosis of tumor cell lines	1.16E-07	1.15E-04	-2.57	Lesion formation	2.50E-04	2.92E-02	-1.353
Degradation of protein	2.63E-03	4.00E-02	-2.57	Concentration of lipid	2.45E-04	2.92E-02	-1.137
Necrosis	3.15E-07	2.48E-04	-2.54	Concentration of fatty acid	6.70E-03	4.48E-02	-1.091
Cell death	1.46E-10	5.75E-07	-2.49	Activation of macrophages	1.26E-02	5.59E-02	-1.091
Inflammatory response	2.21E-03	3.55E-02	-2.47	Size of lesion	2.94E-03	3.36E-02	-0.747
Accumulation of myeloid cells	7.89E-03	5.22E-02	-2.42	Size of bone	1.20E-02	5.44E-02	-0.682
Colony formation of tumor cell lines	1.49E-03	2.79E-02	-2.39	Proliferation of cells	1.24E-02	3.36E-02	-0.578
Proliferation of tumor cells	7.12E-03	5.22E-02	-2.35	Concentration of triacylglycerol	1.45E-03	4.93E-02	-0.412
Synthesis of reactive oxygen species	2.91E-04	1.00E-02	-2.35	Quantity of reactive oxygen species	8.63E-03	4.89E-02	-0.283
Colony formation of cells	3.24E-04	1.07E-02	-2.30				
Accumulation of neutrophils	5.54E-04	1.45E-02	-2.20				
Killing of bacteria	3.49E-04	1.12E-02	-2.19				
Generation of reactive oxygen species	9.50E-04	2.04E-02	-2.13				
Atrophy of muscle	2.97E-03	4.19E-02	-2.11				
Differentiation of connectivetissue	1.24E-03	2.46E-02	-2.05				
Metastasis of melanoma celllines	1.47E-04	6.57E-03	-2.00				
IBxDU^3^							
Quantity of erythroid progenitor cells	2.14E-03	3.55E-02	2.19	Organismal death	5.13E-03	4.13E-02	2.615
Cytolysis	2.99E-05	2.73E-03	2.18	Necrosis of epitelial tissue	3.53E-03	3.72E-02	1.450
Engulfment of cells	3.67E-03	4.74E-02	2.05	Metabolism of reactive oxygen species	1.09E-02	5.44E-02	1.261
Morphology of cells	7.37E-05	4.53E-03	2.05	Neuronal cell death	8.40E-03	4.93E-02	1.202
Mean corpuscular hemoglobin concentration	4.14E-04	1.22E-02	2.00	Transport of molecule	1.21E-02	5.44E-02	1.154
Cellviability of tumor celllines	4.72E-03	5.22E-02	1.99	Oxidation of lipid	2.74E-04	3.02E-02	1.127
Extension of neurites	3.92E-03	4.94E-02	1.98	Celldeath of epithelialcells	4.45E-03	4.13E-02	1.118
Cell viability	8.69E-05	4.96E-03	1.97	Colonyformation of tumor celllines	1.39E-02	5.96E-02	1.103
Quantity of reticulocytes	1.99E-03	3.36E-02	1.96	Hydrolysis of triacylglycerol	7.45E-06	7.39E-03	1.091
Cytosis	5.19E-03	5.22E-02	1.94	Morbidity or mortality	1.15E-02	5.44E-02	1.067
Cell survival	3.18E-05	2.78E-03	1.86	Inflammation of body cavity	6.07E-03	4.13E-02	1.000
Anemia	1.58E-05	1.81E-03	1.80	Cell death	5.47E-04	3.36E-02	0.980
Phagocytosis of cells	1.49E-03	2.79E-02	1.80	Cancer	7.95E-03	4.93E-02	0.825
Hyperplasia of epidermis	8.90E-05	5.00E-03	1.71	Hypertrophy	1.08E-02	5.44E-02	0.808
Size of cells	8.43E-03	5.22E-02	1.60	Cell death of central nervous system cells	7.74E-03	4.93E-02	0.791
Binding of cells	2.60e-03	3.97E-02	1.49	Mineralization of cells	1.06E-04	1.81E-02	0.762
Binding of granulocytes	2.43e-04	8.61E-03	1.47	Apoptosis	3.24E-03	3.47E-02	0.731
Mass of muscle	1.96e-03	4.53E-02	1.38	Inflammation of organ	2.73E-03	3.36E-02	0.712

^1^ IB = Purebred Iberian pigs

^2^ Z-score: Predicted activation status of biological function. The higher the value, the more activated the functions is predicted to be.

^3^ IBxDU = Iberian x Duroc crossbred pigs

#### Effect of muscle type, *Biceps femoris* or *Longissimus dorsi*, on gene expression

Differences in gene expression were observed between LD and BF muscles, 83 genes showing higher expression levels in LD than in BF muscle (FC from 1.7 to 27.0), while 52 genes were upregulated in BF muscle (FC from 1.8 to 183.2) (p < 0.01, FDR < 0.123). Genes such as *HOXA11*, *PVALB (Parvalbumin)* or *CXCL13* (upregulated in BF muscle) and *IBSP*, *ZIC1* and *MMP13* (upregulated in LD muscle) showed the largest expression differences (S7 Table).

IPA software was used to detect enriched pathways (S8 Table) and biological functions (S9 Table) associated with the DE genes between both muscles. Moreover, the regulators study identified 370 regulatory genes. Among them, 233 were identified by IPA software, 143 were identified following the RIFs approach and 10 were found DE between developmental stages (S10 Table). SIM1 was identified in the three different analysis carried out, which highlights its importance and potential role in regulating muscle gene expression in IB and IBxDU pigs.

In order to validate the results obtained from the RNA-Seq analysis, the relative expression of five DE genes affected by the genetic type, the growing stage or the studied muscle was assessed by qPCR in all the available samples. All of them were validated by qPCR (S11 Table). A concordance correlation coefficient, used to assess technical validation in high throughput transcriptomic studies [36] was calculated (CCC = 0.94) and denoted a high general concordance between RNA-Seq and qPCR expression values. In general good individual correlation values were obtained (S11 Table).

## Discussion

### Phenotypic results

#### Effect of developmental stage and terminal sire line on Iberian pig phenotype

Developmental stage affected carcass characteristics, as expected: all measured parameters increased along time (Table 2). On the other hand, genetic type affected all the carcass phenotypic parameters in newborns: IBxDU were bigger and heavier (p < 0.001) than IB piglets (Table 2). However, growing IB and IBxDU pigs only differed in ham weight and circumference, but no significant difference was found in other body size measures. Differences in ham measures between genotypes observed in juvenile pigs agree with results obtained in pure and Duroc-crossbred Iberian pigs at final slaughter weight [40, 41]. Previous studies also reported that adult Duroc-crossbred Iberian pigs are longer and heavier than their purebred Iberian counterparts [40, 41], but these differences in body weight and size between genotypes are not evident at weaning [29]. Iberian pigs have a lower prenatal growth potential than lean pigs [42]. Thus, the finding of IB newborns being smaller in body size and weight than IBxDU is expected, due to the effect of the leaner and more growth-efficient Duroc sire. Nevertheless, the lack of body weight differences between genotypes at weaning and growing stages is unexpected.

Iberian pigs show some special characteristics, associated with the phenomenon known as *thrifty genotype*, which might led to differences in voluntary feed intake and energy expenditure between IBxDU and IB pigs [29].Such differences may lead to a compensatory gain in the latter during the suckling period, which would not be reflected on adult pigs due to a much lower growth potential of pure Iberian animals in later stages. However, this hypothesis cannot be tested under the design of the present experiment and further studies specially designed to analyze the evolution of growth and energy balance in different genotypes along early and juvenile growth would be required.

Regarding biochemical parameters, plasma glucose levels were higher in neonates, probably because growing animals were slaughtered after a fasting period, which may deplete plasma glucose levels. At growing, IBxDU pigs showed higher plasma glucose levels than IB pigs, in agreement with the reported decreased plasma glucose levels in obese when compared to lean fasted pigs, probably because of a high rate of glucose utilization for fat synthesis in obese pigs [43]. Fructosamine levels were higher in older pigsin agreement with previous studies [44].

Certain lipid metabolism-related parameters (cholesterol, HDL and triglycerides) were significantly different between IB and IBxDU newborns, but seemed to balance in growing pigs, with a significant interaction observed between developmental stage and genotype for these parameters: newborn IB pigs showed greater plasma cholesterol, HDL and triglycerides than IBxDU piglets [30], but these differences disappeared over time. The evolution over life span in lipid related plasma indicators has not been previously compared between different pig breeds. However, in a study assessing plasma biochemical parameters in wild boars, those under 6 months of age showed slightly higher cholesterol and doubled triglyceride levels than wild boars over 6 months of age [45], while an increase as pigs mature in the concentrations of cholesterol has been reported in lean pigs [46]. Thus, a different regulation of lipid metabolism in swine breeds over life might be responsible for the observed differences in cholesterol, HDL and triglycerides, in agreement with previous findings. Also, the stability of lipid metabolism-related parameters may be associated with a state of ‘healthy’ obesity (without metabolic disorders) that purebred Iberian pigs can maintain under intensive production, whereas crossbred Iberian pigs might accumulate more cholesterol due to the concentrated-based diet provided during the starter and growing production stages.

Intramuscular fat content was also affected by pigs’ growth:total IMF content increased over time (p < 0.0001) in both genetic types (Table 2). In newborns, no differences in loin IMF content were observed between IBxDU and IB pigs, in contrast with results reported for muscle BF of newborn IB piglets, which showed almost 30% more IMF than BF of IBxDU pigs [30]. This supports that regulation of IMF deposition depends, among other factors, on muscle type [47, 48]. However, growing IB pigs showed greater IMF content than growing IBxDU pigs in LD muscle, in concordance with the characteristic difference between genetic types in adult animals [13].

Regarding IMF composition, MUFA content increased over time (p < 0.0001) in both genetic types. The increase in MUFA content was due to an increase in the most abundant fatty acid, oleic acid (C18:1 n-9). Regarding the genetic type effect on IMF composition, a slight difference was observed between genetic types at birth, while a stronger effect was observed at growth. The most remarkable effect was an increase in n-3 fatty acids and a decrease in the ratio n-6/n-3 in IB when compared to IBxDU growing pigs. A decrease in the ratio n-6/n-3 and increase of DPA and DHA fatty acids may promote consumers’ health [49–51]. These results indicate that crossing with Duroc sires decreased meat quality in terms of consumers’ health and IMF concentration in LD muscle of growing pigs, in agreement with differences observed in weaned and adult pigs [13, 29].

#### Effect of muscle type on intramuscular fat content and composition

We observed higher PUFA content in BF when compared to LD muscle, which was in part due to increased n-3 PUFA content in BF. This also led to a decrease in the ratio n-6/n-3. It is known that the pattern of fatty acids deposition may differ across muscles [15, 18, 52]. In agreement with the results of the present study, the biggest difference previously observed between LD and BF muscle of lean pigs was reported for PUFA content [26], which might be due to the differences in oxidative properties observed between muscles [19, 20]. As previously discussed, a lower ratio n-6/n-3 has been associated with healthier meat. Moreover, when analyzing jointly the data of both muscles, the inclusion of Duroc on Iberian genetics significantly increased SFA content in muscle of IBxDU newborns (Table 3), in accordance with previous results [13]. High SFA consumption increases cardiovascular disease risk in human [53]. Thus, meat from BF muscle, the main muscle within the ham and from pure Iberian pigs, could be considered healthier than meat from LD muscle and from Duroc-crossbred Iberian pigs.

### Transcriptome analysis

Three main effects (developmental stage, muscle type and genetic type at birth and at the growing stages) were evaluated in the present study.

These effects were assessed in two muscles and time points. *Longissimus dorsi* and BF muscle were assessed because they represent the most important pork cuts, the loin and the ham. On the other hand, the two time points were selected due to the interest of comparing the initial (birth) and an intermediate (four months of age) stages during the growing period, which in traditional Iberian pig production is considered up to eight months of age [4].

**Fig 1:**
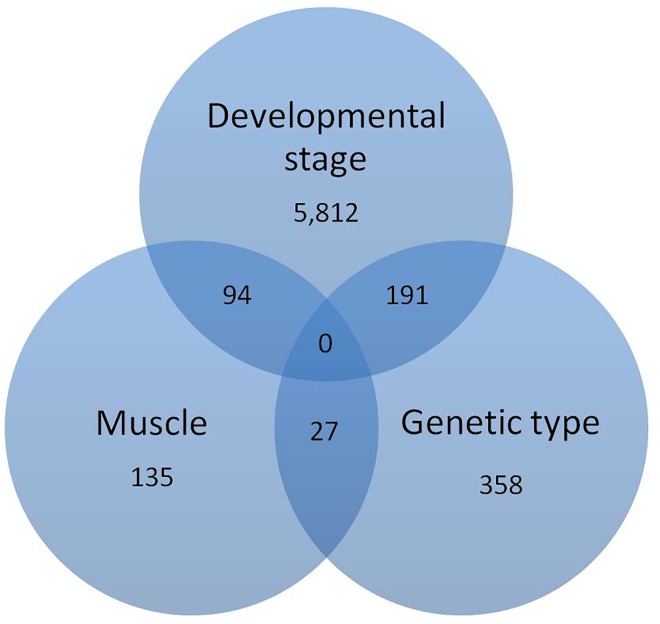
Venn diagram showing common differentially expressed genes across studied conditions. Developmental stage, the biggest studied effect, shows several common differentially expressed genes with other effects, while expression of any gene was affected by the three studied effects.

Several genes showed changes in expression according to more than one of the studied effects (Fig 1). Developmental stage affected a high number of genes which were concomitantly affected by muscle or genetic type, probably due to its large impact on muscle transcriptome. However, few common genes were observed between muscle and genetic type effects and no gene was observed to be affected across the three studied conditions. Fourteen common genes (nine of them being known genes) were affected by genetic type in both newborn and growing pigs, and will be further discussed because of their potential phenotypic impact.

#### Effect of developmental stage on *Longissimus dorsi* transcriptome

The developmental stage was the main factor affecting gene expression in pure and Duroc-crossbred Iberian pigs, as 5,800 genes changed their expression levels between both developmental stages. Gene expression has been previously reported to change across age in pigs, especially during early stages of prenatal and postnatal development [54]. Some of the genes showing the highest upregulation in newborn piglets were involved in the development of different tissues, such as bone, cartilage, adipose or muscle tissues (*ACTC1*, *ARHGAP36*, *IBSP*, *TNN*, *ATP6V0D2*, *COMP*, *FGF21*, *DLK1* and several myosin and collagen proteins). Moreover, two genes associated with meat quality were highly upregulated at birth: *RETN* is associated with adipogenesis and lipogenesis and *TNN* is involved in the development of the extracellular matrix in pigs [55, 56]. Genes highly upregulated in growing pigs were associated with the immune response (since several immunoglobulin genes such as *IGLC*, *IGLV7*, *8*, *9* and *10*, were identified), but also with protein metabolism (*PVALB*, *UBD*). A functional interpretation of the DE genes upregulated in each growing period was carried out. Several metabolic pathways were enriched at both developmental stages, most of them involved in muscle growth (*Wnt/β-catenin Signaling*, *Calcium Signaling*, *Signaling by Rho Family GTPases or Actin Cytoskeleton Signaling*), in agreement with the studied tissue and growth period. Pathways enriched in newborn piglets are involved in cholesterol, triglycerides and other compounds biosynthesis, characteristic of a highly proliferative developmental stage [57, 58]. This is concordant with other enriched functions related to cellular growth and anabolic processes (as *invasion of cells*, *transport of molecule*, *cell movement*, *adhesion of connective tissue cells*, *proliferation of fibro blasts* and *synthesis of carbohydrate*). Contrarily, normal non-proliferating or differentiated cells primarily utilize nutrients to fuel basic cellular processes and predominantly mediate catabolic metabolism to efficiently generate ATP [57, 58], as observed in growing pigs. At this age, enriched pathways were mainly related to catabolic processes (*Protein Ubiquitination Pathway*, *Glycolysis I*, *Gluconeogenesis I*, *Glucocorticoid Receptor Signaling* and *Phospholipase C Signaling*) andbiological functions represented a decrease in developmental and growth processes. Moreover, functions such as *contractility of skeletal muscle*, *mass of muscle*, *quantity of muscle cells* and *function of muscle*, appeared enriched in growing pigs; this suggests a more developed and functional muscular system in older than in newborn piglets [54].

Changes in gene expression and thus, phenotypic consequences, between developmental stages were predicted to be regulated by a number of TRFs. Identified regulators affecting muscle development include the myogenesis regulators *MYOD1*, *MEF2C* and *MEF2D*, essential ones at different stages of muscle growth [54, 59, 60]. The forkhead family members *FOXO1* and *FOXO3* play a role in muscle but also in adipose tissue development [61–64], while *PPARG*, *PPARGC1B*, *SIM1*, *ATF4* and *CEBPD* regulate adipocyte differentiation, energy homeostasis and lipid metabolism [65–69]. Common genes identified by the different followed procedures were of special interest. Specifically, *HSF1*, *JARID2*, *TCF4* and *YBX3* play known roles in muscle development and protein metabolism [70–74], while *TFEB* and *MYC* are involved in adipocyte proliferation, lipid metabolism and fatty acid transport [75, 76]. These TRF potentially regulate muscle and fat accretion in young pigs and are thus, of special interest in the understanding of molecular mechanisms underlying such processes in pigs and other species.

#### Effect of genetic type on *Longissimus dorsi* transcriptome

Genetic type significantly affected gene expression over pig´s growth. Nine known genes (*GPT2*, *PSAT1*, *ART5*, *ADAMTS8*, *KCNH2*, *RASSF9*, *TP63*, *ENV* and *ASB5*) were found DE between genotypes at both developmental stages, suggesting an important role of these genes. Interestingly, three of them (*GPT2*, *PSAT1* and *ADAMTS8*) were differentially regulated at both ages, being upregulated in IBxDU at birth and in IB at four months. *GPT2* is involved in gluconeogenesis, fatty acids oxidation and amino acid metabolism [77, 78], while *PSAT1* catalyzes serine biosynthesis. *ADAMTS8* has an important role in inhibition of angiogenesis [79]. Expression changes observed in these genes, related to muscle growth and metabolism, may suggest a differential muscle growth regulation depending on developmental stage and genetic type, and are in agreement with the observed phenotypic results.

Differentially expressed genes and enriched pathways and functions were of special interest when related to processes that may drive phenotypic differences observed between IB and IBxDU pigs. We found two main processes that seemed to be affected by the genetic type:

Muscle growth:

Genes showing strong upregulation in newborn IBxDU pigs were associated with the extracellular matrix structure (*MATN1* and 3 or *COL9A1* and 2), connective tissue and muscle development (*GDF5*, *MYH10*) and also with protein metabolism and degradation (*PVALB*, *HSPs*). *MYH10* was also upregulated in IbxDU pigs in two previous studies comparing muscle transcriptome of newborn [30] and weaning [29] IB and IbxDU pigs, indicating a relevant role for this gene in muscle development differences between genotypes. These results are concordant with the greater prenatal development and with the enriched pathways (Table 5) in crossbred newborns.

**Fig 2:**
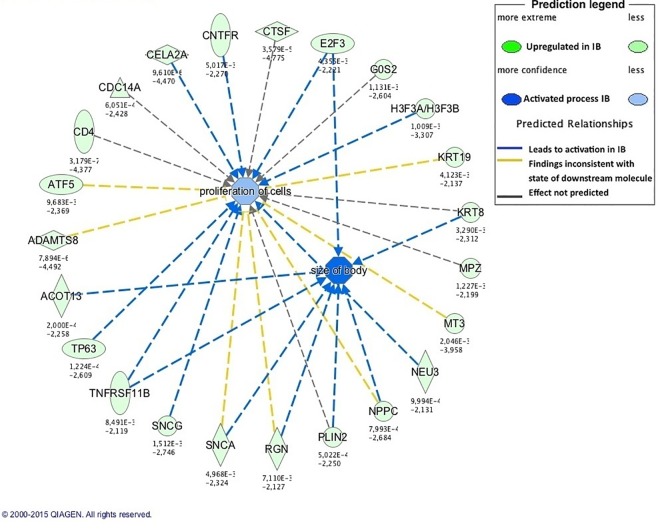
Enriched biological functions related to body growth in growing Iberian (IB) pigs. The network generated by IPA software shows enriched biological functions in IB pigs (blue color) and genes predicted to be involved in enrichment of these functions. The activation of cell proliferation and body size in growing IB pigs might be related to a compensatory growth occurring in those pigs when compared to crossbred (IBxDU) pigs during the early growing period.

In growing pigs, pathways involved in the metabolism of non-essential amino acids such as serine, glycine and alanine were enriched in the IB genotype. Those amino acids are necessary for synthesis of proteins and other biomolecules needed for cell proliferation [80], suggesting an active protein synthesis in growing IB pigs. In accordance with this, several genes involved in *body size* and *cell proliferation* were upregulated in IB pigs (Fig 2), probably associated with the increased body growth that the smaller IB neonates could have developed, leading to the observed similar body weight in growing IB and IbxDU pigs.

**Fig 3:**
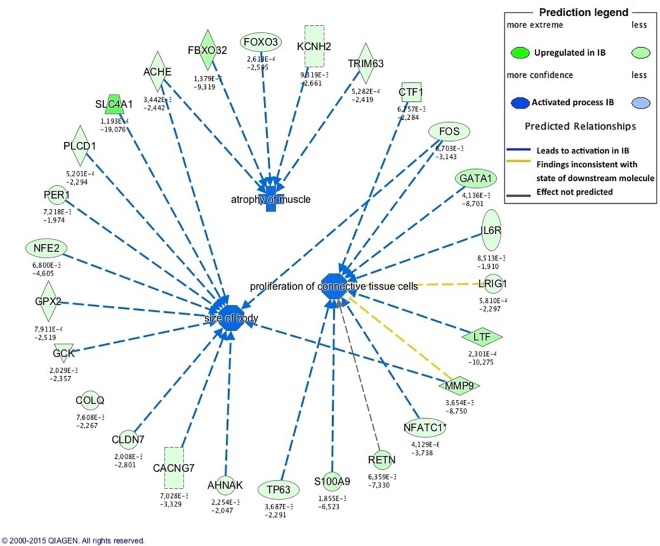
Enriched biological functions related to growth and development in newborn Iberian (IB) piglets. The activation of muscle atrophy and the upregulation of genes involved in protein metabolism and degradation in IB pigs suggests a more active protein turnover in these pigs when compared to crossbred (IBxDU) pigs. Functions associated with animal growth were also enriched in IB newborn pigs.

Muscle growth is not only determined by cell proliferation, but also by protein synthesis and degradation and angiogenesis. Protein degradation seems to be an active process in both IB and IbxDU newborn piglets. In the present study, IbxDU pigs showed upregulation of genes (*ELANE*, *MMP9*, *FBXO32*, *PVALB*, *HSPS1*, *HSPA4L* and *DNAJA1*) and pathways (Table 4) related to protein degradation, although enrichment of muscle degradation or atrophy functions was observed in IB (Fig 3) but not in IbxDU pigs (Table 5), similarly to the results observed in BF muscle [30]. This suggests greater muscle degradation in newborn IB than IbxDU pigs [81]. Accordingly, genes showing high upregulation in four months-old IB pigs were mainly related to protein turnover and degradation (*CTSF*, *ADAMTS8* or *CELA2*).

Energy homeostasis, inflammation and immune system:

Energy homeostasis is tightly regulated in animals due to its importance for normal growth and survival. Pathways involved in cholesterol (*LXR/RXR Activation*, *Atherosclerosis Signaling*) and glucose metabolism (*Glucocorticoid Receptor Signaling*, *GDP-glucose Biosynthesis and Glucose* and *Glucose-1-phosphate Degradation*) were enriched in both newborn and growing IB pigs, suggesting an increased energy metabolism in pure Iberian piglets.

**Fig 4:**
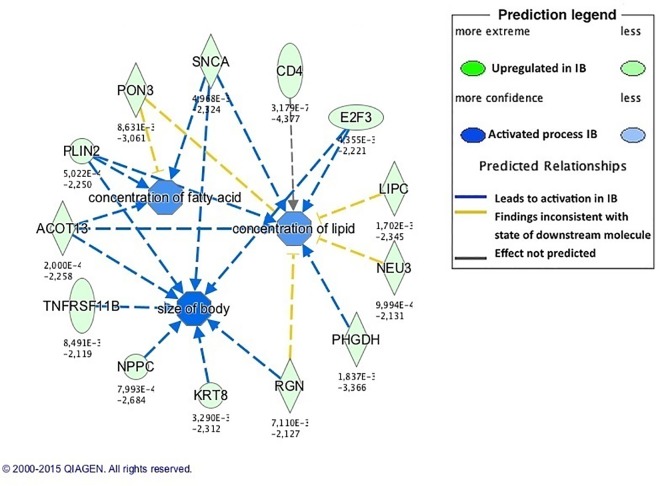
Enriched biological functions related to lipid metabolism in growing Iberian (IB) pigs. The network generated by IPA software shows metabolism-related enriched biological functions in IB pigs. Concentration of lipid seems to be a more active process in IB pigs, in agreement with phenotypic differences observed in loin intramuscular fat content.

A pathway involved in the control of energy homeostasis, cell metabolism and muscle development, the *Wnt/Ca+ signaling pathway* [82] was enriched (p = 0.009) in newborn IB pigs. This pathway included genes upregulated in IB piglets, which were involved in adipogenesis and in the development of obesity, as *NFATC1* and *PLCD1* [83, 84]. In agreement, biological functions related to glucose metabolism and lipid accumulation, as *concentration of lipid*, *concentration of fatty acid* and *concentration of triacylglycerol* (Fig 4), were enriched in growing IB pigs. Moreover, the *LPS/IL-1 Mediated Inhibition of RXR* pathway, enriched in those pigs was reported to positively correlate with fat area [85].

On the other hand, the *glucocorticoid receptor signaling* pathway, was enriched in both IBxDU and IB newborns, which might be related to the farrowing stress and with the wide range of actions associated to this pathway, from catabolic processes to glucose and energy homeostasis or adipocyte differentiation [86, 87]. However, the upregulated genes in IBxDU pigs associated to this pathway are members of the HSPs family, and thus, its activation might be a consequence of cellular stress, or activated protein catabolism. On the other hand, DE genes involved in this pathway in IB pigs play important roles in protein catabolism (FOXO3A, [64]), but also in lipid metabolism and adipogenesis (*NFATC1*, *FOS*, *FOXO3A*, [83, 88, 89]) and in osteogenesis and glucose uptake (*BGLAP*, [90]). Thus, although the *glucocorticoid receptor signaling* pathway was enriched in both IB and IBxDU piglets, a different set of DE genes was involved in each genetic type and thus, different metabolic consequences might be expected.

The juvenile IBxDU pigs showed enrichment of pathways mainly involved in biosynthesis and degradation of retinoids, such as retinoate and retinol or melatonin, involved in the synchronization of the circadian clock, associated with the control of energy homeostasis, among a wide range of functions [91]. The enrichment of these functions is concordant with the upregulation of genes involved in degradation of compounds such as steroids and fatty acids (*CYP1A1*) [92]. The *GABA (Gamma-Aminobutyric Acid Type A) receptor signaling* pathway was also found enriched in growing IBxDU pigs. Beyond its function as an inhibitory neurotransmitter [93], the expression of GABA receptor in human muscle was associated with increased resting energy expenditure [94]. Thus, IBxDU pigs might have a greater basal energy expenditure that would decrease their potential for fat accumulation, in agreement with enriched biological functions and with the lower IMF content observed in IBxDU pigs.

Regulators analysis:

We performed a regulatory factors study to investigate the driving molecular mechanisms responsible for the differences in gene expression observed between genetic types. Three different approaches, based on bibliographic (IPA software) and expression and co-expression (RIFs analysis) information were combined as a powerful strategy to identify overlapping TRFs [30].

A total of 122 TRF were identified in newborn and 62 in older pigs. Among them, 16 TRFs were identified at both developmental stages. These common TRF would be expected to have a deeper impact in the final phenotype and thus, should be considered as strong candidate genes driving phenotypic differences in pure and crossbred Iberian pigs. Some of them, such as *MYOD1*, a well-known myogenic regulator, and some TRF recently associated with its regulation (*BHLHE40* and *HDAC2*; [95, 96]) are necessary for muscle development. Similarly, *CTNNB1 (Catenin Beta 1)* is a component of the *Wnt signaling* pathway, related to cell differentiation and metabolism, including myogenesis and muscle regeneration in adult animals [82]. Also, TRF involved in the immune response (*IRF9*, *NFKBIA*, *REL*) were found to affect gene expression in IB and IBxDU newborn and growing pigs. *NFKBIA* has a critical role in the upregulation of pro-inflammatory factors and is considered a link between immunological stress and obesity [97, 98]. The identification of TRF such as *NFKBIA*, *ATF4* or *CEBPA*, with direct roles in adipogenesis, and fat accumulation [66, 99] in an obese pig breed such as the Iberian pig [29] is of high interest to understand regulatory mechanisms responsible for fatness regulation.

Regarding the developmental stage-specific TRF identified, 15 regulator genes were identified using different approaches at birth. It is noteworthy that some of them (*MEF2C*, *MEF2D*, *MYOG*, *SOX4)* are important TRF involved in muscle cell differentiation [59, 100]. In addition, TRF related to protein degradation (*CREB3L1*, *HSF1* and *CREBBP*) and to blood cells differentiation (*KLF1*, *SF1* and *NFE2*) were also identified and may play a role in muscle development, due to the need of protein degradation and blood supply in the growing muscle. Moreover, *KLF1* was strongly upregulated in IBxDU pigs (25.61), which suggest an important role of this TRF. Other important TRFs identified in the performed analysis were *FOS* and *FOXO1*, involved in muscle differentiation and metabolism [63], but also in the regulation of adipogenic genes (as *PPARG*) expression and IMF accumulation [54, 62]. These TRFs were also previously identified in BF muscle of newborn piglets [30].

In growing pigs, only 3 TRFs (*EN1 (Engrailed Homeobox 1)*, *IRF2 (Interferon Regulatory Factor 2)* and *TCF7L2 (Transcription Factor 7 Like 2)*) were identified simultaneously using different approaches. *EN1* and *IRF2* play important roles in regulating development and cell cycle. In muscle cells, *IRF2* activates transcription of the *VCAM-1* gene, suggested to play a role in the differentiation of skeletal muscle [101]. *EN1* and *TCF7L2* interact with the *Wnt signaling pathway* [102], which negatively regulates adipogenesis [103]. Variants in *TCF7L2* gene have been reported to affect insulin secretion and body mass index and to promote type II diabetes [104, 105]. Thus, it seems that lipid metabolism might be more tightly regulated in growing IB and IBxDU pigs.

**Fig 5:**
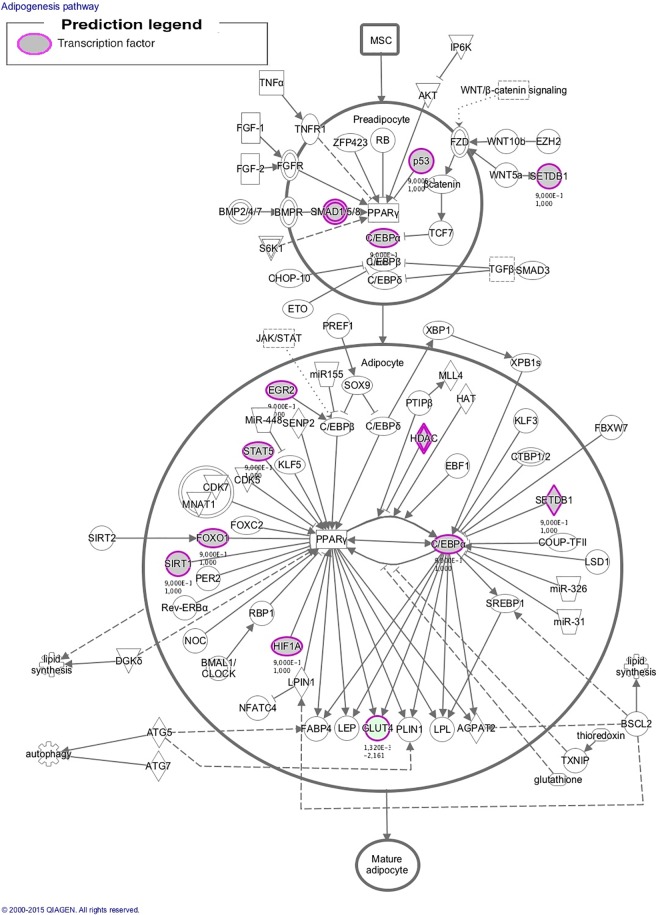
Adipogenesis pathway. Several transcription factor identified in the regulators study and one gene upregulated in Iberian pigs are involved in the adipogenesis pathway, most of them regulate *PPARg* expression or activation.

To better understand the role of the identified regulators on gene expression and on phenotypic differences, information from the DE and regulators analyses was used for biological interpretation, focusing on enriched metabolic pathways (S5 Table). The *glucocorticoid receptor signaling* and the *adipogenesis* (Fig 5) were the most enriched pathways at birth. Changes in regulation and function of *adipogenesis* pathway in newborn piglets may determine the differences in fatness observed in LD muscle of growing IB and IBxDU pigs, in accordance with results observed in BF muscle of newborn piglets [30]. The *glucocorticoid receptor signaling* may be also implicated in such differences, due to its roles in energy homeostasis and adipocyte differentiation [82, 83].

The *aryl hydrocarbon receptor* was the most enriched pathway in growing pigs. This pathway is involved in xenobiotics metabolism, although a role in inhibition of lipid biosynthesis and adipocyte differentiation has also been reported [106].

A total of fifty-one pathways were enriched at both stages. Among them, the *PPAR signaling*, *the aryl hydrocarbon recepto*r, *the adipogenesis*, *the Wnt* and the *unfolded protein response* pathways are particularly interesting because these are specifically involved in adipocyte differentiation and protein degradation. Moreover, finding such pathways at two different growing stages, suggests a long-term activation that may also drive differences in pure and crossbred Iberian pigs in the adulthood.

### Effect of muscular tissue, *Biceps femoris* or *Longissimus dorsi*, on gene expression at birth

Differences in the transcriptomic profile were observed between BF and LD of newborn IB and IBxDU pigs. However the effect was smaller than that reported when comparing LD and *Semimembranosus* muscles [27], probably due to the sampling at an early age, when muscle fiber type is still not well determined [107, 108].

In the present study, 83 genes were upregulated in LD muscle, some of them (*IBSP*, *ZIC1* and *MMP13*) showing large expression differences. *ZIC1* and *MMP13* are implicated in the early control of myogenesis and in myostatin signaling [109–112]. On the other hand, genes highly upregulated in BF muscle are involved in myogenesis control (*HOXA11*), by regulating *MYOD* expression [113], muscle contraction (*PVALB*), or the immune response and adipocyte differentiation, [114]. *PVALB* gene expression was also deeply affected by developmental stage (larger expression in growing pigs) and by genetic type in newborn pigs, being upregulated in both BF and LD muscles of IBxDU pigs [30]. The finding of this DE gene across tissues, developmental stages and genetic types suggests an active role of *PVALB* gene on phenotypic changes.

The biological interpretation retrieved several enriched pathways in LD muscle related to lipid metabolism (*Atherosclerosis Signaling* and *VDR/RXR Activation*) muscle development and function (*Calcium Signaling*, *Inhibition of Matrix Metalloproteases* and *Actin Cytoskeleton Signaling*) and to the immune response (*Agranulocyte Adhesion and Diapedesis*, *Leukocyte Extravasation Signaling*, *Granulocyte Adhesion and Diapedesis* and *IL-8 Signaling*). In agreement with the enriched pathways, several biological functions related to inflammation and immune response were predicted by IPA software to be activated in LD muscle. On the other hand, functions such as *proliferation of cells* or *size of body*, related to cell growth and development were enriched in LD muscle of newborn pigs. In agreement, biological functions (*neovascularization*, *development of cardiovascular system* and *vasculogenesis*) and pathways (HIF1α Signalling) related to the supply of oxygen and energy to growing cells, were also enriched in LD muscle suggesting a more active cellular growth.

**Fig 6:**
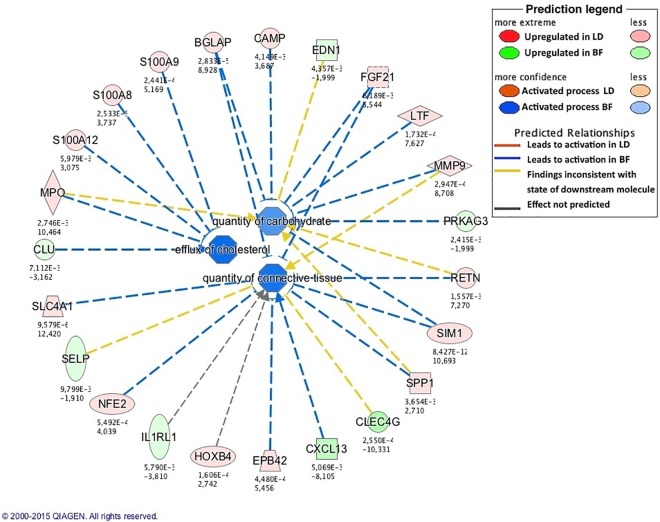
Enriched biological functions in *Biceps femoris* (BF) muscle. The network generated by IPA software shows enriched biological functions in BF muscle, suggesting that lipid metabolism is more active in BF when compared to *Longissimus dorsi* muscle.

On the other hand, BF muscle showed enriched pathways involved in adipocyte differentiation and lipid metabolism (*LXR/RXR Activation*, *VDR/RXR Activation*, *Atherosclerosis Signaling*, *FXR/RXR Activation* and *biosynthesis of retinoids*, *bile acids and thyroid hormone*; [115–118]), in agreement with the enrichment of *efflux of cholesterol* in BF muscle. This suggests a more active lipid metabolism in BF muscle. In agreement, *quantity of connective tissue* and *quantity of carbohydrate* functions were also enriched in BF when compared to LD muscle (Fig 6), probably associated to a different energy homeostasis in both muscles. A more active lipid metabolism in BF muscle might allow a deeper impact of the genotype on lipid deposition, leading to the observed significant differences in IMF content between pig genotypes in BF, which are not evident in LD muscle of neonates. Moreover, these differences in lipid metabolism may be associated with the higher IMF content reported in BF than in LD muscle in adult pigs [47].

Beyond metabolic pathways and biological functions, we performed a regulatory genes search in the same way as for the other main effects. Among the 370 identified regulators, *SIM1* was considered the most significant regulator, as it was highlighted in the three followed approaches. Although no information exists regarding *SIM1* gene effects on pigs, the differential expression across tissues, developmental stages and genetic type in newborn piglets, suggest this TRF as a strong candidate gene. Previous information on its effects on adiposity, energy expenditure and food intake in other species such as mouse or humans [119–122], supports its importance as a regulator of fatness traits.

## Conclusions

The present study reports the effects of developmental stage, muscle and genetic type on animal phenotype, muscle transcriptome, metabolic pathways and transcriptional regulation, associated with traits of interest. Developmental stage represented the most drastic influence on phenotype and transcriptome. Newborn pigs showed enrichment of anabolic functions, while predominant functions in the growing stage were related to catabolism and muscle functioning, indicating a decrease in developmental and growth processes and a more advanced muscle development in juvenile pigs. Moreover, phenotypic differences regarding body size and plasma biochemical parameters were observed between genetic types at birth but dramatically decreased at growing. This suggests strong differences in early growth patterns and metabolism between them, in spite of the closely related analyzed genotypes. In agreement, IMF differences between genotypes also depended on developmental stage and muscle. Gene expression results support the phenotypic findings, as DE genes and pathways suggest a different timing in growth, proliferative and anabolic processes. Those processes were upregulated in IBxDU newborn pigs (associated with a higher capacity for prenatal growth) and in growing IB pigs (in agreement with a potential compensatory gain during the postnatal period). Differences in metabolism were also observed, and results suggest a more active lipid and glucose metabolism in both newborn and growing IB pigs, in agreement with their greater potential for fat accumulation. An effect of muscle type on muscular metabolic characteristics was observed, as BF muscle showed increased lipid metabolism, while LD was characterized by growth and proliferative processes. The regulatory factors analysis identified several remarkable TRF (as *MYOD1*, *NFKBIA*, *FOXO1*, *MEFs*, *TCF7L2*, *SIM1* and *PVALB*), selected due to their identification following different methodological approaches, their identification across different developmental stages and muscles and their potential roles on regulating molecular processes underlying differences in metabolism and productive traits between IB and IBxDU pigs.

## Supporting Information

S1 Table Differentially expressed genes conditional on developmental stage (birth vs growth). (XLSX)

S2 Table Enriched pathways in the set of DE genes conditional on developmental stage (birth vs growth). (XLSX)

S3 Table Transcription factors affecting Longissimus dorsi gene expression differences between newborn and growing Iberian pigs. (XLSX)

S4 Table Differentially expressed genes conditional on genetic type (Iberian (IB) vs Duroc X Iberian (IBxDU)) at birth and at growth. (XLSX)

S5 Table Transcription factors affecting Longissimus dorsi gene expression differences between pure and Duroc-crossbred Iberian pigs at birth and at growing stages. (XLSX)

S6 Table Enriched pathways in the set of differentially expressed genes and transcription factors conditional on genetic type at birth and at growth. (XLSX)

S7 Table Differentially expressed genes conditional on muscle (*Biceps femoris* (BF) vs *Longissimus dorsi* (LD)). (XLSX)

S8 Table Enriched pathways in the set of differentially expressed genes conditional on muscle type (*Longissimus dorsi* (LD) vs *Biceps femoris* (BF)) at birth and growth. (XLSX)

S9 Table Enriched biological functions in the set of differentially expressed genes conditional on muscle type (*Longissimus dorsi* (LD) vs *Biceps femoris* (BF)) at birth and growth. (XLSX)

S10 Table Transcription factors affecting differences in gene expression between *Longissimus dorsi* and *Biceps femoris* muscles from Iberian pigs at birth. (XLSX)

S11 Table RNA-Seq and qPCR validation results and correlation coefficient (r) between the two used methodologies (XLSX)

## References

[1] Chang K, Da Costa N, Blackley R, Southwood O, Evans G, Plastow G, et al Relationships of myosin heavy chain fibre types to meat quality traits in traditional and modern pigs. Meat Science. 2003;64(1):93–103. 22062667 10.1016/s0309-1740(02)00208-5

[2] Gonzalez‐Añover P, Encinas T, Gomez‐Izquierdo E, Sanz E, Letelier C, Torres‐Rovira L, et al Advanced onset of puberty in gilts of thrifty genotype (Iberian pig). Reproduction in domestic animals. 2010;45(6):1003–7. 10.1111/j.1439-0531.2009.01476.x 19473306

[3] Astiz S, Gonzalez‐Bulnes A, Astiz I, Barbero A, Perez‐Solana M, Garcia‐Real I. Advanced onset of puberty after metformin therapy in swine with thrifty genotype. Experimental physiology. 2014;99(9):1241–52. 10.1113/expphysiol.2014.081455 25085845

[4] López-Bote C. Sustained utilization of the Iberian pig breed. Meat Science. 1998;49:S17–S27. 22060709

[5] Ovilo C, Fernández A, Noguera J, Barragán C, Letón R, Rodríguez C, et al Fine mapping of porcine chromosome 6 QTL and LEPR effects on body composition in multiple generations of an Iberian by Landrace intercross. Genet Res. 2005;85(01):57–67. 16089036 10.1017/s0016672305007330

[6] Muñoz G, Ovilo C, Silió L, Tomás A, Noguera J, Rodríguez M. Single-and joint-population analyses of two experimental pig crosses to confirm quantitative trait loci on chromosome 6 and leptin receptor effects on fatness and growth traits. J Anim Sci. 2009;87(2):459–68. 10.2527/jas.2008-1127 18952727

[7] Daza A, Lopez-Bote CJ, Olivares A, Menoyo D, Ruiz J. Age at the beginning of the fattening period of Iberian pigs under free-range conditions affects growth, carcass characteristics and the fatty acid profile of lipids. Anim Feed Sci Technol. 2007;139(1–2):81–91.

[8] Rodriguez-Sanchez JA, Ripoll G, Latorre MA. The influence of age at the beginning of Montanera period on meat characteristics and fat quality of outdoor Iberian pigs. Animal. 2010;4(2):289–94. 10.1017/S1751731109991029 22443883

[9] Čandek-Potokar M, Žlender B, Bonneau M, editors. Effects of breed and slaughter weight on longissimus muscle biochemical traits and sensory quality in pigs. Ann Zootech; 1998: EDP Sciences. 10.1016/s0309-1740(97)00109-5 22063077

[10] Latorre M, Medel P, Fuentetaja A, Lázaro R, Mateos G. Effect of gender, terminal sire line and age at slaughter on performance, carcass characteristics and meat quality of heavy pigs. ANIMAL SCIENCE-GLASGOW THEN PENICUIK-. 2003;77(1):33–46.

[11] Lebret B, Juin H, Noblet J, Bonneau M. The effects of two methods of increasing age at slaughter on carcass and muscle traits and meat sensory quality in pigs. Animal Science. 2001;72:87–94.

[12] Unruh J, Friesen K, Stuewe S, Dunn B, Nelssen J, Goodband R, et al The influence of genotype, sex, and dietary lysine on pork subprimal cut yields and carcass quality of pigs fed to either 104 or 127 kilograms. J Anim Sci. 1996;74(6):1274–83. 8791199 10.2527/1996.7461274x

[13] Ventanas S, Ventanas J, Jurado A, Estevez M. Quality traits in muscle biceps femoris and back-fat from purebred Iberian and reciprocal Iberian x Duroc crossbred pigs. Meat Science. 2006;73(4):651–9. 10.1016/j.meatsci.2006.03.009 22062566

[14] Wood JD, Enser M, Fisher AV, Nute GR, Sheard PR, Richardson RI, et al Fat deposition, fatty acid composition and meat quality: A review. Meat Science. 2008;78(4):343–58. 10.1016/j.meatsci.2007.07.019 22062452

[15] Sharma N, Gandemer G, Goutefongea R. Comparative lipid composition of porcine muscles at different anatomical locations. Meat Science. 1987;19(2):121–8. 10.1016/0309-1740(87)90017-9 22055863

[16] Sepe A, Tchkonia T, Thomou T, Zamboni M, Kirkland JL. Aging and regional differences in fat cell progenitors–a mini-review. Gerontology. 2011;57(1):66–75. 10.1159/000279755 20110661 PMC3031153

[17] Gregoire FM, Smas CM, Sul HS. Understanding adipocyte differentiation. Physiol Rev. 1998;78(3):783–809. Epub 1998/07/23. 9674695 10.1152/physrev.1998.78.3.783

[18] Leseigneur-Meynier A, Gandemer G. Lipid composition of pork muscle in relation to the metabolic type of the fibres. Meat Science. 1991;29(3):229–41. 10.1016/0309-1740(91)90052-R 22061275

[19] Andrés AI, Cava R, Mayoral AI, Tejeda JF, Morcuende D, Ruiz J. Oxidative stability and fatty acid composition of pig muscles as affected by rearing system, crossbreeding and metabolic type of muscle fibre. Meat Science. 2001;59(1):39–47. 22062504 10.1016/s0309-1740(01)00050-x

[20] Karlsson A, Enfält A-C, Essén-Gustavsson B, Lundström K, Rydhmer L, Stern S. Muscle histochemical and biochemical properties in relation to meat quality during selection for increased lean tissue growth rate in pigs. J Anim Sci. 1993;71(4):930–8. 8478293 10.2527/1993.714930x

[21] Li W, Zhao S, Huang Y, Yang M, Pan H, Zhang X, et al Expression of lipogenic genes during porcine intramuscular preadipocyte differentiation. Res Vet Sci. 2012;93(3):1190–4. 10.1016/j.rvsc.2012.06.006 22795880

[22] Pérez-Enciso M, Ferraz AL, Ojeda A, López-Béjar M. Impact of breed and sex on porcine endocrine transcriptome: a bayesian biometrical analysis. BMC Genomics. 2009;10(1):89. 19239697 10.1186/1471-2164-10-89 PMC2656523

[23] Voillet V, SanCristobal M, Lippi Y, Martin PGP, Iannuccelli N, Lascor C, et al Muscle transcriptomic investigation of late fetal development identifies candidate genes for piglet maturity. BMC Genomics. 2014;15. 10.1186/1471-2164-15-797 PMC4287105 25226791

[24] D’Andrea M, Dal Monego S, Pallavicini A, Modonut M, Dreos R, Stefanon B, et al Muscle transcriptome profiling in divergent phenotype swine breeds during growth using microarray and RT‐PCR tools. Anim Genet. 2011;42(5):501–9. 10.1111/j.1365-2052.2010.02164.x 21906101

[25] Zhao Y, Li J, Liu H, Xi Y, Xue M, Liu W, et al Dynamic transcriptome profiles of skeletal muscle tissue across 11 developmental stages for both Tongcheng and Yorkshire pigs. BMC Genomics. 2015;16(1):377. 25962502 10.1186/s12864-015-1580-7 PMC4437458

[26] Sobol M, Krawczyńska A, Skiba G, Raj S, Weremko D, Herman A. The effect of breed and feeding level on carcass composition, fatty acid profile and expression of genes encoding enzymes involved in fat metabolism in two muscles of pigs fed a diet enriched in n-3 fatty acids. A preliminary study. Journal of Animal and Feed Sciences. 2015;648:127.

[27] Herault F, Vincent A, Dameron O, Le Roy P, Cherel P, Damon M. The longissimus and semimembranosus muscles display marked differences in their gene expression profiles in pig. 2014. 10.1371/journal.pone.0096491 PMC4014511 24809746

[28] Te Pas MF, Keuning E, Van De Wiel DJ, Young JF, Oksbjerg N, Kruijt L. Proteome profiles of Longissimus and Biceps femoris porcine muscles related to exercise and resting. Journal of Life Science. 2011;5:598–608.

[29] Óvilo C, Benítez R, Fernández A, Núñez Y, Ayuso M, et al Longissimus dorsi transcriptome analysis of purebred and crossbred Iberian pigs differing in muscle characteristics. BMC genomics. 2014; 15: 413 10.1186/1471-2164-15-413 24885501 PMC4070551

[30] Ayuso M, Fernández A, Núñez Y, Benítez R, Isabel B, Barragán C, et al Comparative Analysis of Muscle Transcriptome between Pig Genotypes Identifies Genes and Regulatory Mechanisms Associated to Growth, Fatness and Metabolism. PloS one. 2015;10(12). 10.1371/journal.pone.0145162 PMC4687939 26695515

[31] Segura J, Lopez-Bote CJ. A laboratory efficient method for intramuscular fat analysis. Food Chem. 2014;145:821–5. Epub 2013/10/17. 10.1016/j.foodchem.2013.08.131 24128551

[32] Lopez-Bote CJ, Rey AI, Isabel B, Sanz R. Effect of feeding diets high in monounsaturated fatty acids and alpha-tocopheryl acetate to rabbits on resulting carcass fatty acid profile and lipid oxidation. Anim Sci. 1997;64:177–86.

[33] Cánovas A, Rincón G, Bevilacqua C, Islas-Trejo A, Brenaut P, Hovey RC, et al Comparison of five different RNA sources to examine the lactating bovine mammary gland transcriptome using RNA-Sequencing. Scientific reports. 2014;4. 10.1038/srep05297 PMC5381611 25001089

[34] Trapnell C, Roberts A, Goff L, Pertea G, Kim D, Kelley DR, et al Differential gene and transcript expression analysis of RNA-seq experiments with TopHat and Cufflinks. Nature protocols. 2012;7(3):562–78. 10.1038/nprot.2012.016 22383036 PMC3334321

[35] Robinson MD, McCarthy DJ, Smyth GK. edgeR: a Bioconductor package for differential expression analysis of digital gene expression data. Bioinformatics. 2010;26(1):139–40. 10.1093/bioinformatics/btp616 19910308 PMC2796818

[36] Miron M, Woody OZ, Marcil A, Murie C, Sladek R, Nadon R. A methodology for global validation of microarray experiments. BMC Bioinformatics. 2006;7(1):333. 16822306 10.1186/1471-2105-7-333 PMC1539027

[37] Reverter A, Hudson NJ, Nagaraj SH, Pérez-Enciso M, Dalrymple BP. Regulatory impact factors: unraveling the transcriptional regulation of complex traits from expression data. Bioinformatics. 2010;26(7):896–904. 10.1093/bioinformatics/btq051 20144946

[38] Hudson NJ, Dalrymple BP, Reverter A. Beyond differential expression: the quest for causal mutations and effector molecules. BMC Genomics. 2012;13(1):356. 22849396 10.1186/1471-2164-13-356 PMC3444927

[39] Almudevar A, Klebanov LB, Qiu X, Salzman P, Yakovlev AY. Utility of correlation measures in analysis of gene expression. NeuroRx. 2006;3(3):384–95. 10.1016/j.nurx.2006.05.037 16815221 PMC3593386

[40] Robina A, Viguera J, Perez-Palacios T, Mayoral AI, Vivo JM, Guillen MT, et al Carcass and meat quality traits of Iberian pigs as affected by sex and crossbreeding with different Duroc genetic lines. Span J Agric Res. 2013;11(4):1057–67.

[41] Serrano M, Valencia D, Nieto M, Lázaro R, Mateos G. Influence of sex and terminal sire line on performance and carcass and meat quality of Iberian pigs reared under intensive production systems. Meat Science. 2008;78(4):420–8. 10.1016/j.meatsci.2007.07.006 22062461

[42] Torres-Rovira L, Tarrade A, Astiz S, Mourier E, Perez-Solana M, De La Cruz P, et al Sex and breed-dependent organ development and metabolic responses in foetuses from lean and obese/leptin resistant swine. PloS one. 2013;8(7):1–9. 10.1371/journal.pone.0066728 PMC3720837 23935823

[43] He Q, Ren P, Kong X, Wu Y, Wu G, Li P, et al Comparison of serum metabolite compositions between obese and lean growing pigs using an NMR-based metabonomic approach. The Journal of nutritional biochemistry. 2012;23(2):133–9. 10.1016/j.jnutbio.2010.11.007 21429726

[44] Torres-Rovira L, Pallares P, Gonzalez-Añover P, Perez-Solana ML, Gonzalez-Bulnes A. The effects of age and reproductive status on blood parameters of carbohydrate and lipid metabolism in Iberian obese sows. Reproductive biology. 2011;11(2):165–71. 21804637 10.1016/s1642-431x(12)60053-9

[45] Casas‐Díaz E, Closa‐Sebastià F, Marco I, Lavín S, Bach‐Raich E, Cuenca R. Hematologic and biochemical reference intervals for Wild Boar (Sus scrofa) captured by cage trap. Veterinary Clinical Pathology. 2015. 10.1111/vcp.12250 25899088

[46] Klem TB, Bleken E, Morberg H, Thoresen SI, Framstad T. Hematologic and biochemical reference intervals for Norwegian crossbreed grower pigs. Veterinary Clinical Pathology. 2010;39(2):221–6. 10.1111/j.1939-165X.2009.00199.x 20051064

[47] Ayuso M, Fernandez A, Isabel B, Rey A, Benitez R, Daza A, et al Long term vitamin A restriction improves meat quality parameters and modifies gene expression in Iberian pigs. J Anim Sci. 2015;93(6):2730–44. 10.2527/jas.2014-8573 26115261

[48] Muriel E, Ruiz J, Ventanas J, Petron MJ, Antequera T. Meat quality characteristics in different lines of Iberian pigs. Meat Science. 2004;67(2):299–307. 10.1016/j.meatsci.2003.11.002 22061327

[49] Simopoulos AP. The importance of the omega-6/omega-3 fatty acid ratio in cardiovascular disease and other chronic diseases. Exp Biol Med. 2008;233(6):674–88. 10.3181/0711-MR-311 18408140

[50] Mozaffarian D, Wu JH. Omega-3 fatty acids and cardiovascular disease: effects on risk factors, molecular pathways, and clinical events. J Am Coll Cardiol. 2011;58(20):2047–67. 10.1016/j.jacc.2011.06.063 22051327

[51] Who J, Consultation FE. Diet, nutrition and the prevention of chronic diseases. World Health Organ Tech Rep Ser. 2003;916(i–viii). 12768890

[52] Kim J, Seong P, Cho S, Park B, Hah K, Yu L, et al Characterization of nutritional value for twenty-one pork muscles. Asian Australasian Journal Of Animal Sciences. 2008;21(1):138.

[53] Simopoulos AP. Omega-6/omega-3 essential fatty acid ratio and chronic diseases. Food Rev Int. 2004;20(1):77–90.

[54] Zhao X, Mo D, Li A, Gong W, Xiao S, Zhang Y, et al Comparative analyses by sequencing of transcriptomes during skeletal muscle development between pig breeds differing in muscle growth rate and fatness. PloS one. 2011;6(5):e19774 10.1371/journal.pone.0019774 21637832 PMC3102668

[55] Kayan A, Cinar M, Uddin M, Phatsara C, Wimmers K, Ponsuksili S, et al Polymorphism and expression of the porcine Tenascin C gene associated with meat and carcass quality. Meat Scencei. 2011;89(1):76–83. 10.1016/j.meatsci.2011.04.001 21530096

[56] Čepica S, Ovilo C, Masopust M, Knoll A, Fernandez A, Lopez A, et al Four genes located on a SSC2 meat quality QTL region are associated with different meat quality traits in Landrace× Chinese‐European crossbred population. Anim Genet. 2012;43(3):333–6. 10.1111/j.1365-2052.2011.02252.x 22486507

[57] DeBerardinis RJ, Mancuso A, Daikhin E, Nissim I, Yudkoff M, Wehrli S, et al Beyond aerobic glycolysis: transformed cells can engage in glutamine metabolism that exceeds the requirement for protein and nucleotide synthesis. Proceedings of the National Academy of Sciences. 2007;104(49):19345–50. 10.1073/pnas.0709747104 PMC2148292 18032601

[58] Vander Heiden MG, Cantley LC, Thompson CB. Understanding the Warburg effect: the metabolic requirements of cell proliferation. science. 2009;324(5930):1029–33. 10.1126/science.1160809 19460998 PMC2849637

[59] Edmondson DG, Cheng T, Cserjesi P, Chakraborty T, Olson EN. Analysis of the myogenin promoter reveals an indirect pathway for positive autoregulation mediated by the muscle-specific enhancer factor MEF-2. Mol Cell Biol. 1992;12(9):3665–77. 1324403 10.1128/mcb.12.9.3665-3677.1992 PMC360220

[60] Ivana L, Ohkawa Y, Berkes CA, Bergstrom DA, Dacwag CS, Tapscott SJ, et al MyoD targets chromatin remodeling complexes to the myogenin locus prior to forming a stable DNA-bound complex. Mol Cell Biol. 2005;25(10):3997–4009. 10.1128/MCB.25.10.3997-4009.2005 15870273 PMC1087700

[61] Allen DL, Unterman TG. Regulation of myostatin expression and myoblast differentiation by FoxO and SMAD transcription factors. American Journal of Physiology-Cell Physiology. 2007;292(1):C188–C99. 10.1152/ajpcell.00542.2005 16885393

[62] Gupta D, Leahy AA, Monga N, Peshavaria M, Jetton TL, Leahy JL. Peroxisome Proliferator-activated Receptor γ (PPARγ) and Its Target Genes Are Downstream Effectors of FoxO1 Protein in Islet β-Cells MECHANISM OF β-CELL COMPENSATION AND FAILURE. J Biol Chem. 2013;288(35):25440–9. 10.1074/jbc.M113.486852 23788637 PMC3757206

[63] Hakuno F, Yamauchi Y, Kaneko G, Yoneyama Y, Nakae J, Chida K, et al Constitutive expression of insulin receptor substrate (IRS)-1 inhibits myogenic differentiation through nuclear exclusion of Foxo1 in L6 myoblasts. PloS one. 2011;6(10):e25655 10.1371/journal.pone.0025655 21991327 PMC3185002

[64] Jaitovich A, Angulo M, Lecuona E, Dada LA, Welch LC, Cheng Y, et al High CO2 Levels Cause Skeletal Muscle Atrophy via AMP-activated Kinase (AMPK), FoxO3a Protein, and Muscle-specific Ring Finger Protein 1 (MuRF1). J Biol Chem. 2015;290(14):9183–94. 10.1074/jbc.M114.625715 25691571 PMC4423704

[65] Tolson KP, Gemelli T, Meyer D, Yazdani U, Kozlitina J, Zinn AR. Inducible neuronal inactivation of Sim1 in adult mice causes hyperphagic obesity. Endocrinology. 2014;155(7):2436–44. 10.1210/en.2013-2125 24773343 PMC4060186

[66] Yu K, Mo D, Wu M, Chen H, Chen L, Li M, et al Activating transcription factor 4 regulates adipocyte differentiation via altering the coordinate expression of CCATT/enhancer binding protein β and peroxisome proliferator‐activated receptor γ. FEBS J. 2014;281(10):2399–409. 10.1111/febs.12792 24673832

[67] Rosen ED, Sarraf P, Troy AE, Bradwin G, Moore K, Milstone DS, et al PPARγ is required for the differentiation of adipose tissue in vivo and in vitro. Mol Cell. 1999;4(4):611–7. 10549292 10.1016/s1097-2765(00)80211-7

[68] Franks PW, Christophi CA, Jablonski KA, Billings LK, Delahanty LM, Horton ES, et al Common variation at PPARGC1A/B and change in body composition and metabolic traits following preventive interventions: the Diabetes Prevention Program. Diabetologia. 2014;57(3):485–90. 10.1007/s00125-013-3133-4 24317794 PMC4154629

[69] Cao Z, Umek RM, McKnight SL. Regulated expression of three C/EBP isoforms during adipose conversion of 3T3-L1 cells. Genes Dev. 1991;5(9):1538–52. 1840554 10.1101/gad.5.9.1538

[70] Londhe P, Davie JK. Interferon-gamma resets muscle cell fate by stimulating the sequential recruitment of JARID2 and PRC2 to promoters to repress myogenesis. Science signaling. 2013;6(305):ra107 Epub 2013/12/12. 10.1126/scisignal.2004633 24327761 PMC4000160

[71] Yasuhara K, Ohno Y, Kojima A, Uehara K, Beppu M, Sugiura T, et al Absence of heat shock transcription factor 1 retards the regrowth of atrophied soleus muscle in mice. J Appl Physiol. 2011;111(4):1142–9. 10.1152/japplphysiol.00471.2011 21817109

[72] Chauhan S, Celi P, Fahri F, Leury B, Dunshea F. Dietary antioxidants at supranutritional doses modulate skeletal muscle heat shock protein and inflammatory gene expression in sheep exposed to heat stress. J Anim Sci. 2014;92(11):4897–908. 10.2527/jas.2014-8047 25349340

[73] Walters ZS, Villarejo-Balcells B, Olmos D, Buist TW, Missiaglia E, Allen R, et al JARID2 is a direct target of the PAX3-FOXO1 fusion protein and inhibits myogenic differentiation of rhabdomyosarcoma cells. Oncogene. 2014;33(9):1148 10.1038/onc.2013.46 23435416 PMC3982124

[74] Saito Y, Nakagami H, Azuma N, Hirata S, Sanada F, Taniyama Y, et al Critical roles of Cold Shock Domain Protein A as an endogenous angiogenesis inhibitor in skeletal muscle. Antioxidants & redox signaling. 2011;15(8):2109–20. 21473684 10.1089/ars.2010.3714

[75] Abdesselem H, Madani A, Hani A, Al-Noubi M, Goswami N, Hamidane HB, et al SIRT1 Limits Adipocyte Hyperplasia Through c-Myc Inhibition. J Biol Chem. 2015:jbc. M115. 675645. 10.1074/jbc.M115.675645 PMC4732199 26655722

[76] Angelini C, Nascimbeni AC, Cenacchi G, Tasca E. Lipolysis and lipophagy in lipid storage myopathies. Biochimica et Biophysica Acta (BBA)-Molecular Basis of Disease. 2016;1862(7):1367–73. 27085974 10.1016/j.bbadis.2016.04.008 PMC4879869

[77] Aagaard-Tillery KM, Grove K, Bishop J, Ke X, Fu Q, McKnight R, et al Developmental origins of disease and determinants of chromatin structure: maternal diet modifies the primate fetal epigenome. Journal of molecular endocrinology. 2008;41(2):91–102. 10.1677/JME-08-0025 18515302 PMC2959100

[78] Marion V, Sankaranarayanan S, de Theije C, van Dijk P, Hakvoort TB, Lamers WH, et al. Hepatic adaptation compensates inactivation of intestinal arginine biosynthesis in suckling mice. 2013. 10.1371/journal.pone.0067021 PMC3681768 23785515

[79] Dunn JR, Reed J, Du Plessis D, Shaw E, Reeves P, Gee A, et al Expression of ADAMTS-8, a secreted protease with antiangiogenic properties, is downregulated in brain tumours. Br J Cancer. 2006;94(8):1186–93. 10.1038/sj.bjc.6603006 16570050 PMC2361255

[80] Possemato R, Marks KM, Shaul YD, Pacold ME, Kim D, Birsoy K, et al Functional genomics reveal that the serine synthesis pathway is essential in breast cancer. Nature. 2011;476(7360):346–50. 10.1038/nature10350 21760589 PMC3353325

[81] Rivera-Ferre M, Aguilera J, Nieto R. Muscle fractional protein synthesis is higher in Iberian than in Landrace growing pigs fed adequate or lysine-deficient diets. The Journal of nutrition. 2005;135(3):469–78. 15735080 10.1093/jn/135.3.469

[82] Sethi J, Vidal-Puig A. Wnt signalling and the control of cellular metabolism. Biochem J. 2010;427:1–17. 10.1042/BJ20091866 20226003 PMC4301310

[83] Neal JW, Clipstone NA. A constitutively active NFATc1 mutant induces a transformed phenotype in 3T3-L1 fibroblasts. J Biol Chem. 2003;278(19):17246–54. 10.1074/jbc.M300528200 12598522

[84] Hirata M, Suzuki M, Ishii R, Satow R, Uchida T, Kitazumi T, et al Genetic defect in phospholipase Cδ1 protects mice from obesity by regulating thermogenesis and adipogenesis. Diabetes. 2011;60(7):1926–37. 10.2337/db10-1500 21617180 PMC3121440

[85] Ponsuksili S, Murani E, Brand B, Schwerin M, Wimmers K. Integrating expression profiling and whole-genome association for dissection of fat traits in a porcine model. J Lipid Res. 2011;52(4):668–78. 10.1194/jlr.M013342 21289033 PMC3284160

[86] Peckett AJ, Wright DC, Riddell MC. The effects of glucocorticoids on adipose tissue lipid metabolism. Metabolism. 2011;60(11):1500–10. 10.1016/j.metabol.2011.06.012 21864867

[87] Kadmiel M, Cidlowski JA. Glucocorticoid receptor signaling in health and disease. Trends Pharmacol Sci. 2013;34(9):518–30. 10.1016/j.tips.2013.07.003 23953592 PMC3951203

[88] Lee K, Buhr J, Hausman GJ, Wright T, Dean R. Expression of c-Fos in subcutaneous adipose tissue of the fetal pig. Mol Cell Biochem. 1996;155(1):31–5. 8717436 10.1007/BF00714330

[89] Wang Y-Z, Huang Y-N, Sun K-Y, Qi J-H, Xiang L. Leptin gene transfer regulates fibromuscular development and lipid deposition in muscles via SIRT1, FOXO3a and PGC-1α in mice in vivo. Int J Mol Med. 2011;28(4):617–23. 10.3892/ijmm.2011.711 21617847

[90] Villafán-Bernal JR, Sánchez-Enríquez S, Muñoz-Valle JF. Molecular modulation of osteocalcin and its relevance in diabetes (Review). Int J Mol Med. 2011;28(3):283–93. 10.3892/ijmm.2011.706 21617842

[91] Fonken LK, Nelson RJ. The effects of light at night on circadian clocks and metabolism. Endocr Rev. 2014;35(4):648–70. 10.1210/er.2013-1051 24673196

[92] Liu J, Sridhar J, Foroozesh M. Cytochrome P450 family 1 inhibitors and structure-activity relationships. Molecules. 2013;18(12):14470–95. 10.3390/molecules181214470 24287985 PMC4216474

[93] Lujan R, Shigemoto R, Lopez-Bendito G. Glutamate and GABA receptor signalling in the developing brain. Neuroscience. 2005;130(3):567–80. 10.1016/j.neuroscience.2004.09.042 15590141

[94] Wu X, Patki A, Lara-Castro C, Cui X, Zhang K, Walton RG, et al Genes and biochemical pathways in human skeletal muscle affecting resting energy expenditure and fuel partitioning. J Appl Physiol. 2011;110(3):746–55. 10.1152/japplphysiol.00293.2010 21109598 PMC3070475

[95] Cho OH, Mallappa C, Hernández‐Hernández JM, Rivera‐Pérez JA, Imbalzano AN. Contrasting roles for MyoD in organizing myogenic promoter structures during embryonic skeletal muscle development. Dev Dyn. 2015;244(1):43–55. 10.1002/dvdy.24217 25329411 PMC4276533

[96] Hsiao S, Huang K, Chang H, Chen S. P/CAF rescues the Bhlhe40-mediated repression of MyoD transactivation. Biochem J. 2009;422:343–52. 10.1042/BJ20090072 19522704

[97] Zhang X, Zhang G, Zhang H, Karin M, Bai H, Cai D. Hypothalamic IKKβ/NF-κB and ER stress link overnutrition to energy imbalance and obesity. Cell. 2008;135(1):61–73. 10.1016/j.cell.2008.07.043 18854155 PMC2586330

[98] Miller MR, Zhang W, Sibbel SP, Langefeld CD, Bowden DW, Haffner SM, et al Variant in the 3′ Region of the IκBα Gene Associated With Insulin Resistance in Hispanic Americans: The IRAS Family Study. Obesity. 2010;18(3):555–62. 10.1038/oby.2009.303 19798070 PMC3992855

[99] Ren W, Guo J, Jiang F, Lu J, Ding Y, Li A, et al CCAAT/enhancer-binding protein α is a crucial regulator of human fat mass and obesity associated gene transcription and expression. BioMed research international. 2014;2014. 10.1155/2014/406909 PMC4022073 24877091

[100] Jang S-M, Kim J-W, Kim D, Kim C-H, An J-H, Choi K-H, et al Sox4-mediated caldesmon expression facilitates differentiation of skeletal myoblasts. J Cell Sci. 2013;126(22):5178–88. 24046453 10.1242/jcs.131581

[101] Jesse TL, LaChance R, Iademarco MF, Dean DC. Interferon regulatory factor-2 is a transcriptional activator in muscle where it regulates expression of vascular cell adhesion molecule-1. The Journal of cell biology. 1998;140(5):1265–76. 9490737 10.1083/jcb.140.5.1265 PMC2132685

[102] Bachar-Dahan L, Goltzmann J, Yaniv A, Gazit A. Engrailed-1 Negatively Regulates β-Catenin Transcriptional Activity by Destabilizing β-Catenin via a Glycogen Synthase Kinase-3β–independent Pathway. Mol Biol Cell. 2006;17(6):2572–80. 10.1091/mbc.E06-01-0052 16571670 PMC1474795

[103] Ross SE, Hemati N, Longo KA, Bennett CN, Lucas PC, Erickson RL, et al Inhibition of adipogenesis by Wnt signaling. Science. 2000;289(5481):950–3. 10937998 10.1126/science.289.5481.950

[104] Florez JC, Jablonski KA, Bayley N, Pollin TI, de Bakker PI, Shuldiner AR, et al TCF7L2 polymorphisms and progression to diabetes in the Diabetes Prevention Program. New Engl J Med. 2006;355(3):241–50. 10.1056/NEJMoa062418 16855264 PMC1762036

[105] Munoz J, Lok KH, Gower BA, Fernandez JR, Hunter GR, Lara-Castro C, et al Polymorphism in the transcription factor 7-like 2 (TCF7L2) gene is associated with reduced insulin secretion in nondiabetic women. Diabetes. 2006;55(12):3630–4. 10.2337/db06-0574 17130514

[106] Alexander DL, Ganem LG, Fernandez-Salguero P, Gonzalez F, Jefcoate CR. Aryl-hydrocarbon receptor is an inhibitory regulator of lipid synthesis and of commitment to adipogenesis. J Cell Sci. 1998;111(22):3311–22. 9788873 10.1242/jcs.111.22.3311

[107] Ashmore C, Addis P, Doerr L. Development of muscle fibers in the fetal pig. J Anim Sci. 1973;36(6):1088–93. 4268264 10.2527/jas1973.3661088x

[108] Picard B, Lefaucheur L, Berri C, Duclos MJ. Muscle fibre ontogenesis in farm animal species. Reprod Nutr Dev. 2002;42(5):415–31. 12537254 10.1051/rnd:2002035

[109] Mizugishi K, Hatayama M, Tohmonda T, Ogawa M, Inoue T, Mikoshiba K, et al Myogenic repressor I-mfa interferes with the function of Zic family proteins. Biochem Biophys Res Commun. 2004;320(1):233–40. 10.1016/j.bbrc.2004.05.158 15207726

[110] Pan H, Gustafsson MK, Aruga J, Tiedken JJ, Chen JC, Emerson CP. A role for Zic1 and Zic2 in Myf5 regulation and somite myogenesis. Dev Biol. 2011;351(1):120–7. 10.1016/j.ydbio.2010.12.037 21211521 PMC3045035

[111] Lei H, Leong D, Smith LR, Barton ER. Matrix metalloproteinase 13 is a new contributor to skeletal muscle regeneration and critical for myoblast migration. American Journal of Physiology-Cell Physiology. 2013;305(5):C529–C38. 10.1152/ajpcell.00051.2013 23761625 PMC3761151

[112] Yang X, Koltes JE, Park CA, Chen D, Reecy JM. Gene Co-Expression Network Analysis Provides Novel Insights into Myostatin Regulation at Three Different Mouse Developmental Timepoints. PloS one. 2015;10(2). 10.1371/journal.pone.0117607 PMC4335066 25695797

[113] Yamamoto M, Kuroiwa A. Hoxa‐11 and Hoxa‐13 are involved in repression of MyoD during limb muscle development. Development, growth & differentiation. 2003;45(5‐6):485–98. 10.1111/j.1440-169x.2003.00715.x 14706073

[114] Kabir SM, Lee E-S, Son D-S. Chemokine network during adipogenesis in 3T3-L1 cells: Differential response between growth and proinflammatory factor in preadipocytes vs. adipocytes. Adipocyte. 2014;3(2):97–106. 10.4161/adip.28110 24719782 PMC3979886

[115] Brandebourg TD, Hu CY. Regulation of differentiating pig preadipocytes by retinoic acid. J Anim Sci. 2005;83(1):98–107. 15583048 10.2527/2005.83198x

[116] Brun PJ, Yang KJZ, Lee SA, Yuen JJ, Blaner WS. Retinoids: Potent regulators of metabolism. BioFactors. 2013;39(2):151–63. 10.1002/biof.1056 23281051 PMC3620893

[117] Bonet M, Ribot J, Felipe F, Palou A. Vitamin A and the regulation of fat reserves. Cellular and Molecular Life Sciences CMLS. 2003;60(7):1311–21. 12943220 10.1007/s00018-003-2290-x PMC11138692

[118] Xia S-F, Duan X-M, Hao L-Y, Li L-T, Cheng X-R, Xie Z-X, et al Role of thyroid hormone homeostasis in obesity-prone and obesity-resistant mice fed a high-fat diet. Metabolism. 2015;64(5):566–79. 10.1016/j.metabol.2014.12.010 25669855

[119] Bonnefond A, Raimondo A, Stutzmann F, Ghoussaini M, Ramachandrappa S, Bersten DC, et al Loss-of-function mutations in SIM1 contribute to obesity and Prader-Willi-like features. J Clin Invest. 2013;123(7):3037–41. 10.1172/JCI68035 23778136 PMC3696559

[120] Swarbrick MM, Evans DS, Valle M, Favre H, Wu SH, Njajou OT, et al Replication and extension of association between common genetic variants in SIM1 and human adiposity. Obesity. 2011;19(12):2394–403. 10.1038/oby.2011.79 21512513 PMC4646950

[121] Ramachandrappa S, Raimondo A, Cali AM, Keogh JM, Henning E, Saeed S, et al Rare variants in single-minded 1 (SIM1) are associated with severe obesity. The Journal of clinical investigation. 2013;123(7):3042 10.1172/JCI68016 23778139 PMC3696558

[122] Holder JL, Zhang L, Kublaoui BM, DiLeone RJ, Oz OK, Bair CH, et al Sim1 gene dosage modulates the homeostatic feeding response to increased dietary fat in mice. Am J Physiol-Endoc M. 2004;287(1):E105–E13. 10.1152/ajpendo.00446.2003 14982752

